# Design of Two Alternative Routes for the Synthesis of Naftifine and Analogues as Potential Antifungal Agents

**DOI:** 10.3390/molecules23030520

**Published:** 2018-02-26

**Authors:** Rodrigo Abonia, Alexander Garay, Juan C. Castillo, Braulio Insuasty, Jairo Quiroga, Manuel Nogueras, Justo Cobo, Estefanía Butassi, Susana Zacchino

**Affiliations:** 1 Grupo de Investigación de Compuestos Heterocíclicos (GICH), Departamento de Química, Universidad del Valle, A. A. 25360 Cali, Colombia; talero22@hotmail.co (A.G.); juan.castillo06@uptc.edu.co (J.C.C.); braulio.insuasty@correounivalle.edu.co (B.I.); jairo.quiroga@correounivalle.edu.co (J.Q.); 2 Escuela de Ciencias Químicas, Facultad de Ciencias, Universidad Pedagógica y Tecnológica de Colombia UPTC, Avenida Central del Norte, A. A. 150003 Tunja, Colombia; 3 Department of Inorganic and Organic Chemistry, Universidad de Jaén, 23071 Jaén, Spain; mmontiel@ujaen.es (M.N.); jcobo@ujaen.es (J.C.); 4 Área de Farmacognosia, Facultad de Ciencias Bioquímicas y Farmacéuticas, Universidad Nacional de Rosario, Suipacha 531, CP 2000 Rosario, Argentina; fefabutassi@hotmail.com (E.B.); szaabgil@citynet.net.ar (S.Z.)

**Keywords:** benzylamines, propiophenone salts, γ-aminoalcohols, Mannich-type reaction, allyamines, naftifine analogues, antifungal activity

## Abstract

Two practical and efficient approaches have been implemented as alternative procedures for the synthesis of naftifine and novel diversely substituted analogues **16** and **20** in good to excellent yields, mediated by Mannich-type reactions as the key step of the processes. In these approaches, the γ-aminoalcohols **15** and **19** were obtained as the key intermediates and their subsequent dehydration catalyzed either by Brønsted acids like H_2_SO_4_ and HCl or Lewis acid like AlCl_3_, respectively, led to naftifine, along with the target allylamines **16** and **20**. The antifungal assay results showed that intermediates **18** (bearing both a β-aminoketo- and *N*-methyl functionalities in their structures) and products **20** were the most active. Particularly, structures **18b**, 1**8c**, and the allylamine **20c** showed the lowest MIC values, in the 0.5–7.8 µg/mL range, against the dermatophytes *Trichophyton rubrum* and *Trichophyton mentagrophytes*. Interesting enough, compound **18b** bearing a 4-Br as the substituent of the phenyl ring, also displayed high activity against *Candida albicans* and *Cryptococcus neoformans* with MIC_80_ = 7.8 µg/mL, being fungicide rather than fungistatic with a relevant MFC value = 15.6 µg/mL against *C. neoformans*.

## 1. Introduction

**Figure 1:**
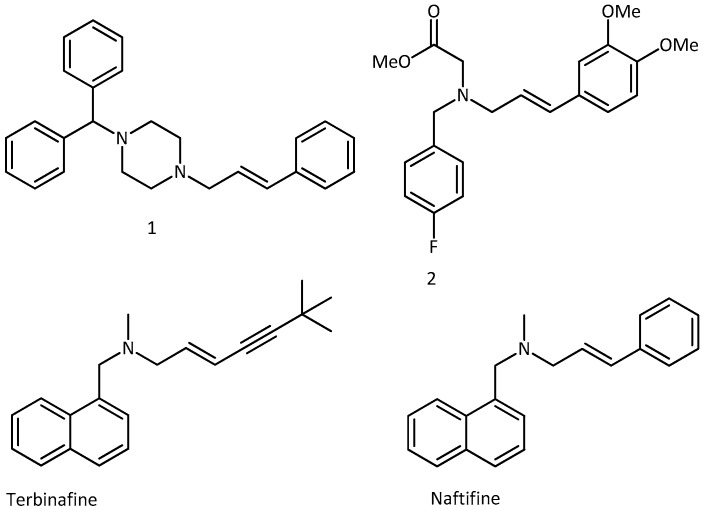
Some allylamines with important biological activities.

Allylamines represent one of the most important primary units in organic chemistry [1]. They are important synthetic precursors for β-aminoacids [2], alkaloids [3] and carbohydrate derivatives [4]. Among the synthetic allylamines of biological importance, it is worth mentioning cinnarizine (**1**), used for the treatment of vertigo and related cerebral disorders [5], abamine (**2**), an important tool to elucidate the mechanisms that regulate the levels of abscisic acid (ABA) in plants and animals [6], terbinafine (brand name Lamisil) and naftifine (brand name Naftin) that are effective antifungal agents. Naftifine is a topical allylamine that is effective against a broad spectrum of dermatophyte fungi of the *Trichophyton* and *Microsporum* spp., and has also shown good activity against *Candida* and *Aspergillus* spp. Terbinafine represents the most effective of this chemical class of antimycotic compounds. Terbinafine proved to be highly active against dermatophytes and *Sporothrix schenckii* and also exerts good activity against several yeasts [7,8,9] (Figure 1).

**Scheme 1:**
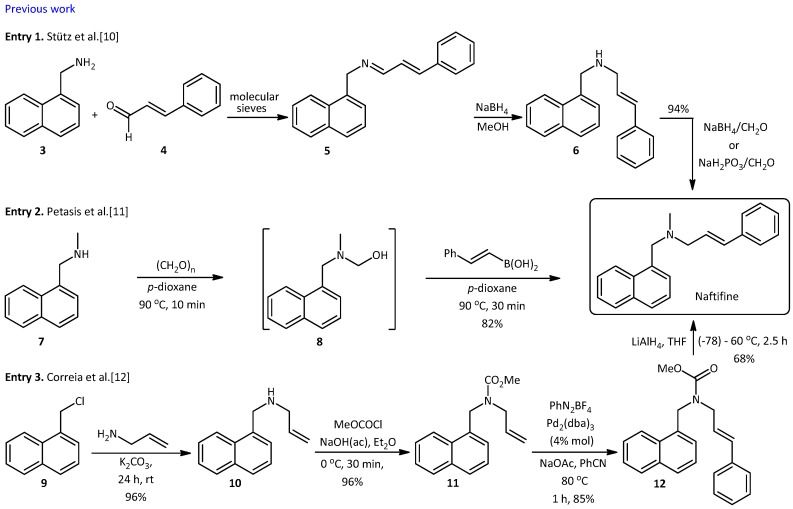
Some previous synthetic approaches for the synthesis of naftifine.

Since naftifine was discovered, different synthetic routes have been developed to obtain it and some of its analogues. Among them, Stütz et al. obtained naftifine in 94% yield via a cinnamyl Schiff´s base (Scheme 1, entry 1) [10], Petasis et al. obtained it in 82% yield via a *trans*-2-phenylvinylboronic acid coupling reaction (Scheme 1, entry 2) [11], and Correia et al. synthesized it in 68% yield via a Heck-type reaction (Scheme 1, entry 3) [12].

Despite the good performance of the allylamines as antifungal agents, there have been some cases in which they have failed in the treatment of patients who have shown antifungal resistance toward some of these drugs [13,14]. These findings suggest the need of looking for new methods and synthesis of new potential antifungal agents inspired in naftifine that can be used as alternatives to the existing ones.

As part of our current program on the synthetic utilization of benzylamine derivatives [15,16,17], herein we wish to report our results on the synthesis of naftifine and analogues through two alternative synthetic strategies mediated in both cases by Mannich-type reactions.

## 2. Results and Discussion

### 2.1. Chemistry

**Scheme 2:**
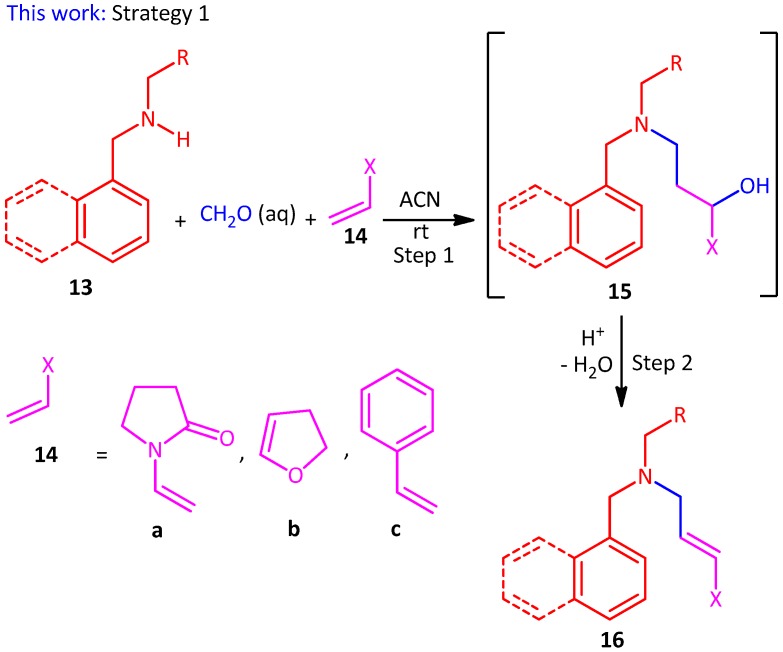
Designed alternative route for the synthesis of naftifine-analogues **16** by combination of a three-component Mannich- and aza-Prins-reactions.

**Scheme 3:**
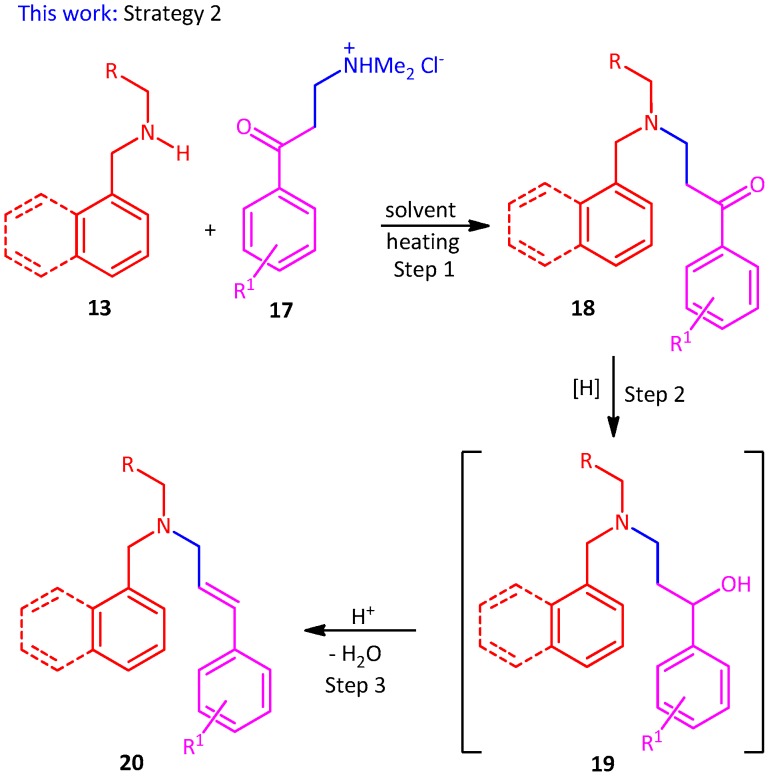
Designed alternative route for the synthesis of naftifine and analogues **20** from a reduction/dehydration strategy.

**Figure 2:**
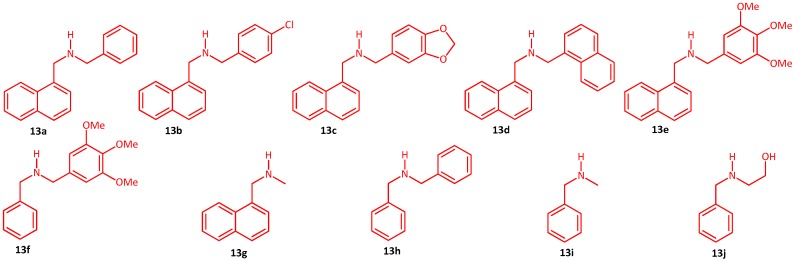
Set of secondary amines **13** used for the synthesis of the intermediate γ-aminoalcohols **15**.

In view of the above, we envisioned that our previous results on the synthesis of γ-aminoalcohols type **15** and **19** [18,19], could be exploited as alternative approaches for the synthesis of naftifine and analogues mediated by Mannich-type reactions. In this direction, two straightforward strategies, shown in Scheme 2 and Scheme 3, were proposed. Strategy 1 consisted on the synthesis of γ-aminoalcohols **15** via a three-component Mannich-type reaction between secondary amines **13**, (see Figure 2), formaldehyde and activated alkenes **14**. Subsequently, a dehydration of **15** should afford the expected allylamines **16**, as shown in Scheme 2. It is remarkable that from Strategy 1, products **16** could be formed by a combination of both Mannich- and aza-Prins-type reactions in a one pot sequence if acid is used as catalyst since Step 1. It is worth mentioning that, the *N*-vinylpyrrolidin-2-one **14a** and 2,3-dihydrofuran **14b** were chosen in this strategy along with styrene **14c** as activated alkenes, due to the fact that these two heterocyclic rings can be found forming part of the structures of synthetic compounds with outstanding antifungal activities [20,21,22,23]. For that reason we supposed that the presence of these heterocycles instead of the phenyl ring in the naftifine analogues (i.e., X = heterocycle in **16**), along with the allylamine moiety in the same structure could produce enhanced effects in the antifungal assays.

The proposed Strategy 2 consisted in the synthesis of γ-aminoalcohols **19** via reduction of their corresponding β-aminoketones **18**. The subsequent dehydration of **19** should also afford a second family of allylamines **20**, as shown in Scheme 3.

Prior to starting, it is worth mentioning that the non-commercial secondary amines **13a**–**f** (Figure 2), were synthesized from their corresponding primary amines and different aryl aldehydes via a reductive amination reaction [15,16,17].

**Scheme 4:**
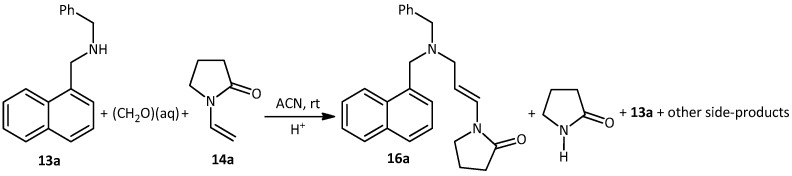
Attempts for acid-catalyzed synthesis of allylamine **16a** via a one-step sequence.

Initially, with the aim of obtaining the allylamines **16**, described in Scheme 2, an acid-catalyzed one-pot approach was planned in order to obtain products **16** directly in a one-step sequence (involving an in situ dehydration), without isolation of their corresponding γ-aminoalcohol intermediates **15** (i.e., from a combination of both Mannich- and aza-Prins-type reactions). Thus, as a model reaction, a mixture of amine **13a** (1.0 mmol), formaldehyde (1.5 mmol) and alkene **14a** (1.0 mmol) in acetonitrile (ACN) was treated with a catalytic amount of conc. H_2_SO_4_ at room temperature for 24 h. The thin-layer chromatography (TLC) analysis showed the formation of a complex mixture of products. Then, the reaction mixture was neutralized with NaOH and their components were separated by column chromatography affording the expected allylamine **16a** in only 11% yield along with unreacted starting amine **13a** and pyrrolidin-2-one (the acid-catalyzed degradation product from **14a**) [24] (Scheme 4).

In an attempt to improve the yield of product **16a** the reaction was repeated with other both stronger (HCl) and milder protic acids (i.e., oxalic, acetic and formic), but unfortunately, with the same behavior and low product yield (Scheme 4). Similar results were obtained when **14a** was replaced by 2,3-dihydrofuran as activated alkene. In a further experiment, the same model reaction depicted in Scheme 4 was performed, but using styrene instead of alkene **14a** in the presence of H_2_SO_4_. After purification and characterization of the product, we were delighted to confirm that naftifine was obtained in an acceptable 65% yield. The higher stability of styrene in acidic medium in comparison with alkene **14a** and 2,3-dihydrofuran (both structures decomposes in such conditions), permitted the selective formation of naftifine in a one-pot procedure (by combination of both Mannich- and aza-Prins-type reactions), as initially was planned in Strategy 1 (Scheme 2).

**Figure 3:**
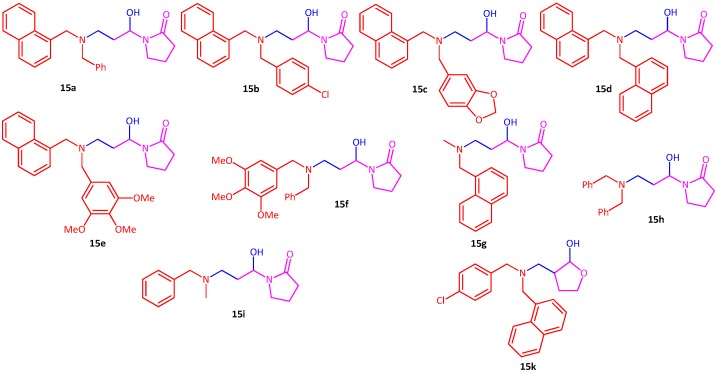
Set of γ-aminoalcohols **15** obtained via a three-component Mannich-type reaction.

Due to the above drawbacks of the acid-catalyzed one-step reaction, when **14a** and 2,3-dihydrofuran were used as activated alkenes, we decided to move the process to a two-step sequence involving the isolation of the γ-aminoalcohol intermediates **15** (Scheme 2). Starting with Step 1 of Strategy 1 depicted in Scheme 2, a set of γ-aminoalcohols **15a**–**k** (Figure 3), was obtained by following our previously established catalyst-free three-component methodology [19]. Thus, a mixture of secondary amines **13** (1.0 mmol, Figure 2), polyformaldehyde (1.5 mmol) and the corresponding activated alkene **14** (1.0 mmol) was stirred at room temperature in ACN to afford the corresponding γ-aminoalcohols **15** in good yields (Figure 3).

**Scheme 5:**
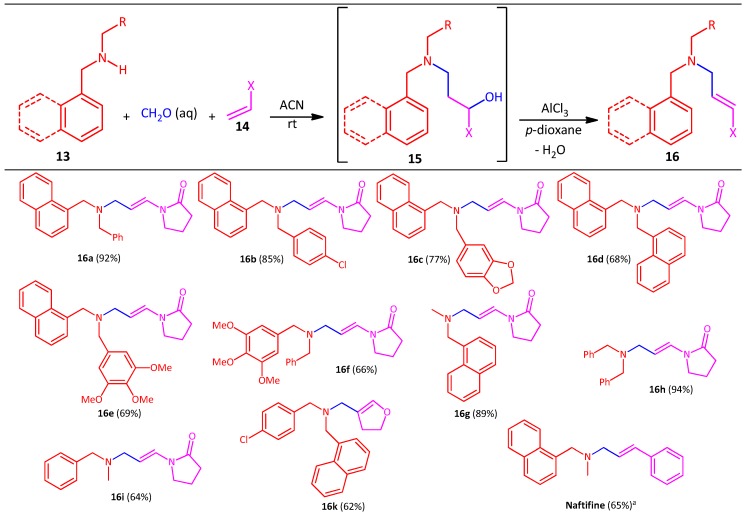
New allylamines **16** obtained by dehydration of the γ-aminoalcohols **15** catalyzed by AlCl_3_. ^a^ Naftifine was obtained from Strategy 1 in a one-pot fashion starting from styrene and catalyzed by sulfuric acid.

Subsequently, after several failed attempts to optimize the dehydration reaction mediated by different Brønsted-Lowry acids (Scheme 2, Step 2), the expected allylamines **16** were obtained by subjecting the corresponding γ-aminoalcohols **15** (1.0 mmol) to reflux in 1,4-dioxane as the solvent in the presence of AlCl_3_ (1.0 mmol) as Lewis acid catalyst. After neutralization with triethylamine (TEA) and purification, the expected compounds **16** were obtained in in acceptable to excellent yields (Scheme 2, Step 2). Scheme 5 depicts the structures of the new allylamines **16** obtained from Strategy 1 (Scheme 2), in a two-step sequence.

**Figure 4:**
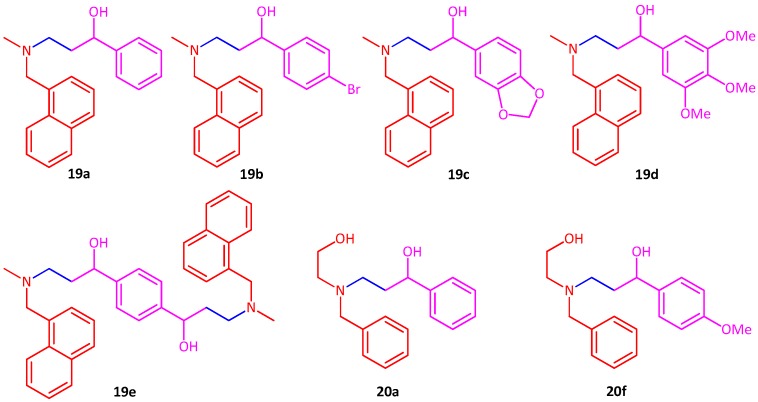
Set of γ-aminoalcohols **19**–**20** obtained via a one-pot S_N_/reduction sequence from amines **13f** and **13h** and propiophenone salts **17a**–**f**.

Regarding Strategy 2, depicted in Scheme 3, we could optimize steps 1 and 2 to obtain satisfactory yields of the expected γ-aminoalcohols **19**–**20** (Figure 4) in a one-pot reaction by treating amines **13** (1.0 mmol) with propiophenone salts **17a**–**f** (1.0 mmol) in a mixture of 1,4-dioxane/TEA at reflux. Compounds **17** were obtained from a Mannich reaction between the corresponding acetophenones, dimethylamine hydrochloride and polyformaldehyde in ethanol at reflux [25]. After removing the solvent, the obtained crude products (corresponding to the β-aminoketones **18**), were reduced by treatment with NaBH_4_ in MeOH affording the expected γ-aminoalcohols **19**–**20** in good to excellent yields (Figure 4).

**Scheme 6:**
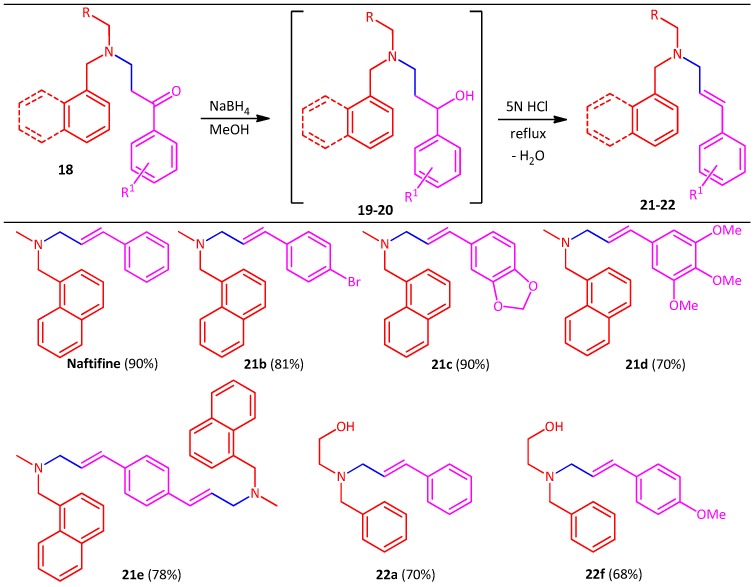
Naftifine and analogues **21**–**22** obtained by dehydration of the γ-aminoalcohols **19**–**20** catalyzed by 5N HCl.

Once we obtained the set of aminoalcohols **19**–**20** (Figure 4), we attempted a couple of trials leading to the expected naftifine and its analogues **21**–**22**. Thus, the expected naftifine and analogues **21**–**22** were obtained in good to excellent yields by refluxing **19**–**20** in 5N HCl (Step 3), followed by neutralization with NaOH and purification. Scheme 6 depicts the structures of the new allylamines **21**–**22**. It is remarkable that naftifine was obtained using Strategy 2 in a very good yield (90%). This value is comparable with the 94% yield obtained previously by Stütz et al., and higher than those obtained through either Petasis or Correia´s methodologies, as shown in Scheme 1. A further advantage of our Strategy 2 is the easy availability of the starting materials used, the simplicity of the processes involved and a major structural diversity in comparison with previous reports.

Finally, structures for all the new compounds obtained from strategies 1 and 2 were fully assigned by IR, NMR, elemental analysis and mass spectra, (see also Supplementary Materials).

### 2.2. Antifungal Activity Studies

Minimum inhibitory concentrations (MIC) of compounds **15**–**22** were determined with the Clinical and Laboratory Standards Institute (CLSI) microbroth dilution methods M27-A3 for yeasts and M38-A2 for filamentous fungi [26,27], against a panel of eight fungal clinically relevant species comprising two yeasts (*Candida albicans* and *Cryptococcus neoformans*), three *Aspergillus* spp. (*A. niger*, *A. fumigatus*, and *A. flavus*) and three dermatophytes (*Trichophyton rubrum*, *T. mentagrophytes* and *Microsporum gypseum*). Compounds were tested at serial two-fold dilutions from 250 to 0.5 μg/mL. Compounds with MICs > 250 μg/mL were considered inactive; between 250–125 μg/mL low active, and in the range 62.5–20 μg/mL, moderately active. MICs below 20 μg/mL was considered as indicative of high activity. From the obtained MIC values of compounds **15**–**22**, some conclusions can be drawn:

(i) In general, the series of precursors **15** and products **16** were significantly less active than the series of precursors **18**–**20** and products **21**–**22**.
(ii) Almost all compounds **15**–**22** showed either very low to moderate activities or were inactive against *A. flavus, A. niger* and *M. gypseum* (data not shown).
(iii) Compounds **15**–**22** identified with the letters **a** and **d**–**k** showed moderate to low (31.2–250 µg/mL) activities against the rest of the fungal panel (data not shown).
(iv) Among compounds **18**–**22**, the structures **18b**, **18c** and **21c** displayed outstanding activities against one or more dermatophytes (0.5–7.8 µg/mL), (Table 1), while **19b** was moderately active (MICs = 31.2–62.5 µg/mL) against four of the fungi tested (i.e., *C. neoformans, M. gypseum, T. rubrum* and *T. mentagrophytes*), being the only compound of the series showing activity against *M. gypseum*. It is worth to take into account that the most active compounds within **18**–**22** possess a CH_3_ group as R substituent although with variations in the substituent R^1^ of the phenyl ring. This finding is in accordance with the required features found by Stütz et al. for the allylamines to display antifungal activity [10].
(v) Compound **18b** was the most active of the whole series, showing activity not only against dermatophytes but also against *Candida* spp., *S. cerevisiae*, *C. neoformans* and *A. flavus* (MICs between 7.8 to 15.6 µg/mL). From these results it is clear that within the compounds bearing 4-Br and 3,4-methylenedioxy R^1^ substituents, the β-aminoketones **18** displayed the best activities, suggesting that the ketone group play an important role in the antifungal activity of these structures. Instead, among the γ-aminoalcohols **19**–**20**, only **19b** showed moderate activity against dermatophytes and *C. neoformans,* while its corresponding allylamine **21b** displayed very low activities (MICs = 125–250 µg/mL) against the whole fungal panel. It is remarkable that allylamine **21c** displayed the most outstanding activities against *T. rubrum* and *T. mentagrophytes* (MICs = 0.5–1.0 µg/mL) constituting this datum a finding that deserves great attention for future research.

It is worth taking into account that compound **18b** displayed high activity against all yeasts as well as *A. fumigatus*. This finding constitutes an interesting result, since previous studies of naftifine-analogues reported no activity against this fungal species [10,28].

**Figure 5:**
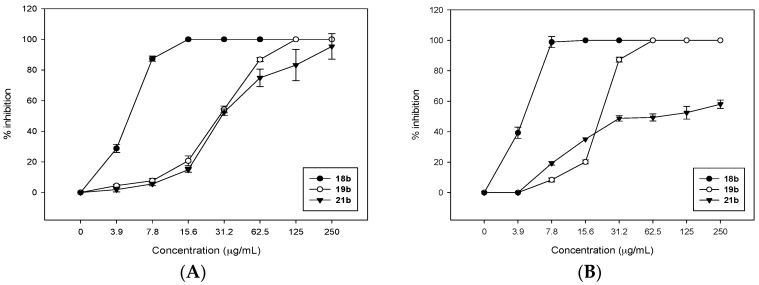
Comparative antifungal activities of compounds **18b**, **19b**, **21b** possessing the same R^1^ substituent but differing in their functional groups (i.e., **18b** is a β-ketoamine; **19b** a γ-aminoalcohol and **21b** an allylamine). (**A**) against *C. albicans*; (**B**) against *C. neoformans*. Amphotericin B (Amp B) inhibits 100% growth at 1.0 µg/mL against *C. albicans* and at 0.5 µg/mL against *C. neoformans*. Curves of Amp B are not included.

In order to have a look into the potential usefulness of **18b** against clinically relevant yeasts, we investigated the fungal inhibition percentages displayed by **18b** against *C. albicans* and *C. neoformans* at concentrations obtained by two fold-dilutions from 250 to 3.9 µg/mL. In addition, the inhibition percentages of **19b** and **21b** were also determined for comparative purposes against the two clinically important fungal species. With these data, two graphs showing inhibition % (Y axis) vs. concentration (X axis), were constructed (Figure 5). The selection of these two fungal species for deepening the studies of the antifungal behavior of **18b**, was due to their clinical relevance. *C. neoformans* is the main cause of cryptococcal meningoencephalitis among HIV patients with impaired defenses that many times led to disease relapse and death [29,30]. In turn, *C. albicans* is the fourth leading cause of nosocomial bloodstream infection (BSI) in intensive care units, causing fatal invasive candidiasis in a high percentage of patients [31]. For these reasons, new compounds that show new potential anticandidal or anti-cryptococcal drugs are highly welcome.

In Figure 5A,B the higher percentage of inhibition of *C. albicans* and *C. neoformans* by **18b** in comparison with **19b** and **21b** can be clearly observed, suggesting that the β-aminoketo structure plays an important role in the anti-yeast activity of this naftifine-analogue. As an example, while **18b** showed 80% inhibition of *C. albicans* at 7.8 µg/mL, **19b** and **21b** inhibited less than 10% and similar results can be observed against *C. neoformans*. Table 2 shows the inhibition percentages values displayed by the above three compounds used to construct Figure 5.

From Table 2 and Figure 5, it is clear that compound **18b** is the most active, being fungicidal rather than fungistatic with a MFC = 15.6 µg/mL against *C. neoformans* and 62.5 µg/mL against *C. albicans*. Instead, **19b** is fungicide at high concentrations (125 and 250 µg/mL, respectively) and **21b** was no fungicide up to 250 µg/mL. In addition, the MIC_80_ of **18b** against both fungi is 7.8 µg/mL (Table 2), a relevant low value that positions **18b** as a good candidate for future research. As it is clearly stated in the CLSI document M27A2 for yeasts, application of a less stringent endpoint such as MIC_80_ (allowing some turbidity above the MIC), has improved inter-laboratory agreement and also discriminates between putatively susceptible and resistant isolates [32].

## 3. Materials and Methods

### 3.1. General Information

Melting points were determined on a Büchi melting point B-450 apparatus (Instrumart, South Burlington, VT, USA) and are uncorrected. FTIR spectra were recorded on a Shimadzu FTIR 8400 spectrophotometer (Scientific Instruments Inc., Seattle, WA, USA) in KBr disks and films. ^1^H- and ^13^C-NMR spectra were recorded on a Bruker Avance 400 spectrophotometer (Bruker BioSpin GmbH, Rheinstetten, Germany) operating at 400 MHz and 100 MHz, respectively, and using CDCl_3_ as solvent and tetramethylsilane as internal standard. DEPT spectra were used for the assignment of carbon signals. Mass spectra were run on a Shimadzu-GCMS 2010-DI-2010 spectrometer (Scientific Instruments Inc., Columbia, SC, USA) (equipped with a direct inlet probe) operating at 70 eV. Microanalyses were performed on a Thermo-Finnigan Flash EA1112 CHN elemental analyzer (Thermo Fischer Scientific Inc., Madison, WI, USA), and the values are within ±0.4% of the theoretical values. Silica gel aluminum plates (Merck 60 F_254_) were used for analytical TLC. The starting chemicals were purchased from (Sigma-Aldrich, San Luis, MO, USA) and Merck Millipore (Billerica, MA, USA) analytical or reagent grade and were used without further purification, unless otherwise noted. All starting materials were weighed and handled in air at room temperature. The reactions were monitored by TLC visualized by a (254/365 nm) UVGL-25 compact UV Lamp (UVP, Upland, CA, USA) and/or with vanillin-H_2_SO_4_ in EtOH. Column chromatography was performed on silica gel (230–400 mesh, Merck). Non-commercially available secondary amines **13a**, **13b**, **13c**, **13d**, **13e** and **13f** were prepared using known procedures [15,16,17,19,33,34].

### 3.2. Synthesis

#### 3.2.1. General Procedure for the Synthesis of Secondary Amines **13a**–**e**

A mixture of primary amine (1.0 mmol) and the appropriate aldehyde (1.0 mmol) was heated in an oil bath at 120 °C for 20–45 min. After a complete disappearance of the starting materials, as monitored by TLC, the mixture was allowed to cool to ambient temperature and dissolved in methanol (4–5 mL). Then, solid NaBH_4_ (2.0 mmol) was added portionwise with stirring over a period of 5 min. The stirring was continued at ambient temperature for 30 min further. After the reaction was complete (monitored by TLC), the volume of the reaction mixture was reduced to 1 mL under reduced pressure, and water (5 mL) was added. The aqueous solution was extracted with EtOAc (2 × 5 mL), and the combined organic layers were dried with anhydrous Na_2_SO_4_. The mixture was filtered and the solvent was removed under reduced pressure. All amines **13** were used without further purification.

#### 3.2.2. General Procedure for the Synthesis of γ-Aminoalcohols **15**

A mixture of secondary amine **13** (~200 mg, 1.0 mmol), polyformaldehyde (1.5 mmol) and the activated alkene **14** (1.1 mmol) was dissolved in ACN (2 mL). The solution was stirred at room temperature for 3 days until the starting secondary amine **13** was no longer detected by TLC (revealed with an ethanolic solution of vanillin-sulfuric acid or iodine). After the excess of solvent was removed under reduced pressure, the oily material obtained was purified by column chromatography on silica gel, using EtOAc:hexane (2:1 *v/v*) as eluent. When the same reaction was performed starting from *N*-methyl-1-(naphthalen-1-yl)methanamine **13a**, polyformaldehyde and styrene in the presence of conc. H_2_SO_4_ (1 drop) as catalyst, during 24 h, afforded directly naftifine as the main reaction product.

#### 3.2.3. General Procedure for the Synthesis of Allylamines **16**

A mixture of the γ-aminoalcohol **15** (200 mg, 1.0 mmol), AlCl_3_ (1.0 mmol) and ACN (4 mL) was stirred at reflux during 2–3 h. After reaction finished (TLC control), TEA (0.5 mL) was added at room temperature. The solvent was removed under reduced pressure, water (5 mL) was added and the aqueous solution was extracted with EtOAc (3 × 5 mL). The combined organic layers were dried with anhydrous Na_2_SO_4_, the mixture was filtered and the solvent was removed under reduced pressure. Finally, the crudes were purified by column chromatography on silica gel using CHCl_3_:MeOH (40:1 *v/v*) as eluent.

*(E)-1-(3-(Benzyl(naphthalen-1-ylmethyl)amino)prop-1-en-1-yl)pyrrolidin-2-one* (**16a**). Following the strategy 1 for the formation of allylamines, the reaction of γ-aminoalcohol **15a** (200 mg, 0.52 mmol) and AlCl_3_ (69 mg, 0.52 mmol) in 4.0 mL of ACN afforded product **16a** as a yellow oil. Yield: 92% (177 mg). FT-IR (film): 3058, 3030, 2925, 2798, 1702, 1660, 1408, 1364, 1334, 1119 cm^−1^. ^1^H-NMR *δ* (ppm): 8.28–8.21 (m, 1H), 7.89–7.82 (m, 1H), 7.77 (d, *J* = 8.0 Hz, 1H), 7.56 (d, *J* = 6.8 Hz 1H), 7.53–7.46 (m, 2H), 7.46–7.41 (m, 1H), 7.37–7.28 (m, 4H), 7.27–7.21 (m, 1H), 7.02 (d, *J* = 14.4 Hz, 1H), 5.00 (dt, *J* = 7.2, 14.4 Hz, 1H), 4.04 (s, 2H), 3.66 (s, 2H), 3.41 (t, *J* = 7.2 Hz, 2H), 3.19 (d, *J* = 6.8 Hz, 2H), 2.46 (t, *J* = 8.2 Hz, 2H), 2.06 (tt, *J* = 7.6, 7.6 Hz, 2H). ^13^C-NMR *δ* (ppm): 173.0 (C=O), 139.7 (Cq), 135.2 (Cq), 133.8 (Cq), 132.4 (Cq), 129.0, 128.3, 128.1, 127.7, 127.3, 126.8, 126.2, 125.5, 125.5, 125.2, 124.8, 108.8, 58.4, 56.6, 54.1, 45.2, 31.2, 17.3. Anal. Calcd. for C_25_H_26_N_2_O: C, 81.05; H, 7.07; N, 7.56. Found: C, 80.82; H, 7.25; N, 7.32.

*(E)-1-(3-((4-Chlorobenzyl)(naphthalen-1-ylmethyl)amino)prop-1-en-1-yl)pyrrolidin-2-one* (**16b**). Following the strategy 1 for the formation of allylamines, the reaction of γ-aminoalcohol **15b** (215 mg, 0.51 mmol) and AlCl_3_ (68 mg, 0.51 mmol) in 4.0 mL of ACN afforded product **16b** as a yellow oil. Yield: 85% (176 mg). FT-IR (film): 3046, 2925, 2882, 2803, 1702, 1660, 1488, 1408, 1298, 1089 cm^−1^. ^1^H-NMR *δ* (ppm): 8.25–8.20 (m, 1H), 7.87–7.83 (m, 1H), 7.77 (d, *J* = 8.4 Hz, 1H), 7.54–7.46 (m, 3H), 7.44–7.39 (m, 1H), 7.24 (s, 4H), 7.01 (d, *J* = 14.4 Hz, 1H), 4.96 (dt, *J* = 7.2, 14.4 Hz, 1H), 4.00 (s, 2H), 3.58 (s, 2H), 3.43 (t, *J* = 7.2 Hz, 2H), 3.15 (d, *J* = 6.8 Hz, 2H), 2.48 (t, *J* = 8.4 Hz, 2H), 2.09 (tt, *J* = 8.0, 8.0 Hz, 2H). ^13^C-NMR *δ* (ppm): 173.0 (C=O), 138.3 (Cq), 134.9 (Cq), 133.8 (Cq), 132.4 (Cq), 132.3 (Cq), 130.2, 128.4, 128.2, 127.8, 127.3, 126.3, 125.5, 125.5, 125.2, 124.7, 108.4, 57.6, 56.8, 54.3, 45.2, 31.2, 17.4. Anal. Calcd. for C_25_H_25_ClN_2_O: C, 74.15; H, 6.22; N, 6.92. Found: C, 74.02; H, 6.50; N, 7.05.

*(E)-1-(3-((Benzo[d][1,3]dioxol-5-ylmethyl)(naphthalen-1-ylmethyl)amino)prop-1-en-1-yl)pyrrolidin-2-one* (**16c**). Following the strategy 1 for the formation of allylamines, the reaction of γ-aminoalcohol **15c** (216 mg, 0.50 mmol) and AlCl_3_ (68 mg, 0.51 mmol) in 4.0 mL of ACN afforded product **16c** as a yellow oil. Yield: 77% (159 mg). FT-IR (film): 3045, 2977, 2922, 2884, 2802, 1701, 1660, 1487, 1441, 1409, 1364, 1298, 1243, 1113, 1038 cm^−1^. ^1^H-NMR *δ* (ppm): 8.30–8.24 (m, 1H), 7.88–7.82 (m, 1H), 7.77 (d, *J* = 8.4 Hz, 1H), 7.57–7.46 (m, 3H), 7.43 (t, *J* = 7.6 Hz, 1H), 7.03 (d, *J* = 14.4 Hz, 1H), 6.86 (s, 1H), 6.80–6.71 (m, 2H), 5.92 (s, 2H, OCH_2_O), 5.00 (dt, *J* = 7.2, 14.4 Hz, 1H), 4.00 (s, 2H), 3.53 (s, 2H), 3.42 (t, *J* = 7.2 Hz, 2H), 3.16 (d, *J* = 6.8 Hz, 2H), 2.46 (t, *J* = 8.0 Hz, 2H), 2.05 (tt, *J* = 7.6, 7.6 Hz, 2H). ^13^C-NMR *δ* (ppm): 172.9 (C=O), 147.4 (Cq), 146.3 (Cq), 135.1 (Cq), 133.7 (Cq), 133.5 (Cq), 132.3 (Cq), 128.2, 127.6, 127.2, 126.1, 125.4 (2 × CH), 125.1, 124.7, 121.9, 109.2, 108.5, 107.7, 100.7, 57.9, 56.4, 53.8, 45.1, 31.1, 17.2. Anal. Calcd. for C_26_H_26_N_2_O_3_: C, 75.34; H, 6.32; N, 6.76. Found: C, 75.48; H, 6.21; N, 6.83.

*(E)-1-(3-((Naphthalen-1-ylmethyl)(napthalen-2-ylmethyl)amino)prop-1-en-1-yl)pyrrolidin-2-one* (**16d**). Following the strategy 1 for the formation of allylamines, the reaction of γ-aminoalcohol **15d** (237 mg, 0.54 mmol) and AlCl_3_ (73 mg, 0.55 mmol) in 4.0 mL of ACN afforded product **16d** as a yellow oil. Yield: 68% (154 mg). FT-IR (film): 3045, 2927, 2882, 2800, 1701, 1660, 1509, 1409, 1363, 1299, 1261, 1224, 1115 cm^−1^. ^1^H-NMR *δ* (ppm): 8.02 (d, *J* = 8.4 Hz, 2H), 7.83 (d, *J* = 8.0 Hz, 2H), 7.77 (d, *J* = 8.4 Hz, 2H), 7.51 (d, *J* = 6.8 Hz, 2H), 7.47–7.38 (m, 4H), 7.28–7.22 (m, 2H), 7.06 (d, *J* = 14.4 Hz, 1H), 5.04 (dt, *J* = 7.2, 14.4 Hz, 1H), 4.04 (s, 4H), 3.42 (t, *J* = 7.2 Hz, 2H), 3.22 (d, *J* = 7.2 Hz, 2H), 2.47 (t, *J* = 8.0 Hz, 2H), 2.07 (tt, *J* = 7.6, 7.6 Hz, 2H). ^13^C-NMR *δ* (ppm): 172.9 (C=O), 135.1 (Cq), 133.8 (Cq), 132.5 (Cq), 128.2, 127.9 (2 × CH), 126.4, 125.4, 125.3, 125.2, 125.1, 108.4, 57.2, 54.6, 45.2, 31.1, 17.4. Anal. Calcd. For C_29_H_28_N_2_O: C, 82.82; H, 6.71; N, 6.66. Found: C, 82.90; H, 6.85; N, 6.48.

*(E)-1-(3-((Naphthalen-1-ylmethyl)(3,4,5-trimethoxybenzyl)amino)prop-1-en-1-yl)pyrrolidin-2-one* (**16e**). Following the strategy 1 for the formation of allylamines, the reaction of γ-aminoalcohol **15e** (239 mg, 0.50 mmol) and AlCl_3_ (68 mg, 0.51 mmol) in 4.0 mL of ACN afforded product **16e** as a yellow oil. Yield: 69% (159 mg). FT-IR (film): 3042, 2298, 2936, 2831, 1700, 1660, 1591, 1505, 1461, 1414, 1230, 1125, 1009 cm^−1^. ^1^H-NMR *δ* (ppm): 8.36–8.31 (m, 1H), 7.87–7.81 (m, 1H), 7.76 (d, *J* = 8.4 Hz, 1H), 7.52–7.37 (m, 4H), 7.05 (d, *J* = 14.4 Hz, 1H), 6.49 (s, 2H), 4.99 (dt, *J* = 7.2, 14.4 Hz, 1H), 4.03 (s, 2H), 3.80 (s, 3H), 3.76 (s, 6H), 3.54 (s, 2H), 3.43 (t, *J* = 7.2 Hz, 2H), 3.22 (d, *J* = 6.8 Hz, 2H), 2.47 (t, *J* = 8.2 Hz, 2H), 2.08 (tt, *J* = 8.0, 8.0 Hz, 2H). ^13^C-NMR *δ* (ppm): 173.0 (C=O), 152.9 (Cq), 136.5 (Cq), 135.8 (Cq), 135.2 (Cq), 133.9 (Cq), 132.4 (Cq), 128.4, 127.9, 127.5, 126.3, 125.4, 125.3, 125.1, 125.0, 108.3, 105.5, 60.7, 58.1, 57.1, 55.9, 54.5, 45.1, 31.1, 17.3. Anal. Calcd. For C_28_H_32_N_2_O_4_: C, 73.02; H, 7.00; N, 6.08. Found: C, 73.15; H, 6.89; N, 6.23.

*(E)-1-(3-Benzyl(3,4,5-trimethoxybenzyl)amino)prop-1-en-1-yl)pyrrolidin-2-one* (**16f**). Following the strategy 1 for the formation of allylamines, the reaction of γ-aminoalcohol **15f** (236 mg, 0.55 mmol) and AlCl_3_ (77 mg, 0.58 mmol) in 4.0 mL of ACN afforded product **16f** as a yellow oil. Yield: 66% (149 mg). FT-IR (film): 2942, 2838, 1704, 1659, 1594, 1166, 1124, 1034, 1009 cm^–1^. ^1^H-NMR *δ* (ppm): 7.37 (d, *J* = 6.8 Hz, 2H), 7.32 (t, *J* = 7.5 Hz, 2H), 7.24 (td, *J* = 1.9, 7.2 Hz, 1H), 7.04 (d, *J* = 14.5 Hz, 1H), 6.62 (s, 2H), 4.95 (dt, *J* = 7.0, 14.3 Hz, 1H), 3.87 (s, 6H), 3.83 (s, 3H), 3.59 (s, 2H), 3.53 (s, 2H), 3.46 (t, *J* = 7.2 Hz, 2H), 3.14 (dd, *J* = 1.8, 6.8 Hz, 2H), 2.48 (t, *J* = 8.2 Hz, 2H), 2.13–2.05 (m, 2H). ^13^C-NMR *δ* (ppm): 173.0 (C=O), 153.1 (Cq), 139.6 (Cq), 136.7 (Cq), 135.5 (Cq), 128.7, 128.2, 126.8, 126.2, 108.6, 105.4, 60.8, 58.1, 57.9, 56.1, 53.9, 45.2, 31.2, 17.4. MS (70 eV, EI): *m*/*z* (%) 409 [M-1]^+^ (5), 300 (12), 124 (100), 181 (39), 91 (45) [PhCH_2_]^+^. Anal. Calcd. For C_24_H_30_N_2_O_4_: C, 70.22; H, 7.37; N, 6.82. Found: C, 70.35; H, 7.23; N, 6.93.

*(E)-1-(3-Methyl(naphthalen-1-ylmethyl)amino)prop-1-en-1-yl)pyrrolidin-2-one* (**16g**). Following the strategy 1 for the formation of allylamines, the reaction of γ-aminoalcohol **15g** (178 mg, 0.57 mmol) and AlCl_3_ (80 mg, 0.60 mmol) in 4.0 mL of ACN afforded product **16g** as a yellow oil. Yield: 89% (149 mg). FT-IR (film): 3045, 2976, 2942, 2878, 2785, 1701, 1660, 1406, 1337 cm^−1^. ^1^H-NMR *δ* (ppm): 8.28 (d, *J* = 8.3 Hz, 1H), 7.86 (d, *J* = 8.0 Hz, 1H), 7.79 (dd, *J* = 1.4, 7.7 Hz, 1H), 7.57–7.51 (m, 1H), 7.51–7.46 (m, 1H), 7.46–7.39 (m, 2H), 7.08 (d, *J* = 14.4 Hz, 1H), 5.06 (dt, *J* = 7.2, 14.4 Hz, 1H), 3.91 (s, 2H), 3.50 (t, *J* = 7.2 Hz, 2H), 3.18 (d, *J* = 7.3 Hz, 2H), 2.49 (t, *J* = 8.4 Hz, 2H), 2.25 (s, 3H), 2.08 (tt, *J* = 7.6, 7.6 Hz, 2H). ^13^C-NMR *δ* (ppm): 173.0 (C=O), 134.8 (Cq), 133.8 (Cq), 132.3 (Cq), 128.3, 127.8, 127.3, 126.1, 125.7, 125.4, 125.0, 124.4, 108.7, 59.8, 58.0, 45.1, 42.2, 31.1, 17.3. Anal. Calcd. For C_19_H_22_N_2_O: C, 77.52; H, 7.53; N, 9.52. Found: C, 77.67; H, 7.64; N, 9.41.

*(E)-1-(3-(Dibenzylamino)prop-1-enyl)pyrrolidin-2-one* (**16h**). Following the strategy 1 for the formation of allylamines, the reaction of γ-aminoalcohol **15h** (172 mg, 0.51 mmol) and AlCl_3_ (69 mg, 0.52 mmol) in 4.0 mL of ACN afforded product **16h** as a yellow oil. Yield: 94% (154 mg) (lit. [19], yellow oil).

*(E)-1-(3-(Benzyl(methyl)amino)prop-1-enyl)pyrrolidin-2-one* (**16i**). Following the strategy 1 for the formation of allylamines, the reaction of γ-aminoalcohol **15i** (134 mg, 0.51 mmol) and AlCl_3_ (69 mg, 0.52 mmol) in 4.0 mL of ACN afforded product **16i** as a yellow oil. Yield: 64% (80 mg). FT-IR (film): 2927, 2885, 1701, 1665, 1589 cm^−1^. ^1^H-NMR *δ* (ppm): 7.31–7.22 (m, 5H), 7.01 (d, *J* = 14.6 Hz, 1H), 5.01 (dt, *J* = 7.2, 14.8 Hz, 1H), 3.51 (t, *J* = 7.8 Hz, 2H), 3.49 (s, 2H), 3.06 (d, *J* = 7.3 Hz, 2H), 2.48 (t, *J* = 8.2 Hz, 2H), 2.18 (s, 3H), 2.15–2.07 (m, 2H). ^13^C-NMR *δ* (ppm): 173.1 (C=O), 138.9 (Cq), 129.1, 128.2, 127.0, 126.3, 108.7, 61.7, 57.5, 45.2, 41.9, 31.2, 17.4. MS (70 eV, EI): *m/z* (%) 243 [M-1]^+^ (7), 153 (63), 124 (100), 120 (26), 91 (56) [PhCH_2_]^+^, 69 (21). Anal. Calcd. For C_15_H_20_N_2_O: C, 73.74; H, 8.25; N, 11.47. Found: C, 73.85; H, 8.12; N, 11.62.

*N-(4-Chlorobenzyl)-1-(4,5-dihydrofuran-3-yl)-N-(naphthalen-1-ylmethyl)methanamine* (**16k**). Following the strategy 1 for the formation of allylamines, the reaction of γ-aminoalcohol **15k** (217 mg, 0.57 mmol) and AlCl_3_ (80 mg, 0.60 mmol) in 4.0 mL of ACN afforded product **16k** as a yellow oil. Yield: 62% (128 mg). FT-IR (film): 3045, 2921, 2888, 2853, 2799, 1663, 1489, 1090 cm^-1^. ^1^H-NMR *δ* (ppm): 8.26–8.21 (m, 1H), 7.90–7.85 (m, 1H), 7.79 (d, *J* = 8.0 Hz, 1H), 7.57 (d, *J* = 6.8 Hz, 1H), 7.53–7.48 (m, 2H), 7.47–7.42 (m, 1H), 7.28–7.26 (m, 4H), 6.29 (s, 1H), 4.35 (t, *J* = 9.4, Hz, 2H), 4.02 (s, 2H), 3.59 (s, 2H), 3.12 (s, 2H), 2.62 (t, *J* = 9.0 Hz, 2H). ^13^C-NMR *δ* (ppm): 143.0, 138.2 (Cq), 135.0 (Cq), 133.8 (Cq), 132.4 (Cq), 132.3 (Cq), 130.1, 128.4, 128.2, 127.7, 126.9, 125.5, 125.4, 125.2, 124.4, 112.3 (Cq), 70.3, 57.6, 56.7, 50.2, 31.8. Anal. Calcd. For C_23_H_22_ClNO: C, 75.92; H, 6.09; N, 3.85. Found: C, 76.05; H, 5.99; N, 4.01.

#### 3.2.4. General Procedure for the Synthesis of the γ-Aminoalcohols **19** and **20**

*(i) Synthesis of the β-aminoketones* **18**. A mixture of amine **13** (500 mg, 1.0 mmol) and the suitable 3-(*N*,*N*-dimethylamino)propiophenone hydrochloride **17** (1.0 mmol) was dissolved in a mixture of 1,4-dioxane (5 mL) and TEA (1 mL). The solution was stirred at reflux for 0.5–2 h until the starting materials were not further detected by TLC. After cooling, the solvent was removed under reduced pressure and the crude was extracted from an aqueous solution with EtOAc (2 × 5 mL). The combined organic layers were dried with anhydrous Na_2_SO_4_ and the solvent was removed under reduced pressure. Ketones **18** were used without further purification for the reduction step.

*(ii) Synthesis of the γ-aminoalcohols* **19** *and* **20**. Residue of the β-aminoketone **18** was re-dissolved in methanol (5 mL) and subjected to reduction by following a similar procedure than the above described for the synthesis of the starting secondary amines **13**. After reaction was completed (TLC control), the crude was purified by column chromatography on silica gel, using a mixture of CH_2_Cl_2_:MeOH (20:1) as eluent.

*3-(Methyl(naphthalen-1-ylmethyl)amino)propan-1-one* (**18a**). Following the general procedure for the formation of β-aminoketones, the reaction of *N*-methyl-1-(naphthalen-1-yl)methanamine (**13g**, 453 mg, 3.01 mmol) and 1-(phenyl)-3-(*N,N*-dimethylamino)propan-1-one hydrochloride (**17a**, 750 mg, 3.51 mmol) in a mixture of 1,4-dioxane (5.0 mL) and TEA (1.0 mL) afforded compound **18a** as an orange solid (168 mg, 21% yield). M.p. = 88–90 °C (amorphous) (lit. [10], 55%).

*1-(4-Bromophenyl)-3-(methyl(naphthalen-1-ylmethyl)amino)propan-1-one* (**18b**). Following the general procedure for the formation of β-aminoketones, the reaction of *N*-methyl-1-(naphthalen-1-yl)methanamine **13g** (515 mg, 3.01 mmol) and 1-(4-bromophenyl)-3-(*N,N*-dimethylamino)propan-1-one hydrochloride (**17b**, 875 mg, 2.99 mmol) in a mixture of 1,4-dioxane (5.0 mL) and TEA (1.0 mL) afforded compound **18b** as a yellow solid (518 mg, 45% yield). M.p. = 55–57 °C (amorphous). FTIR (KBr): 3060, 2947, 2840, 2794, 1685 (C=O), 1584, 1069 cm^−1^. ^1^H-NMR *δ* (ppm): 8.23–8.19 (m, 1H), 7.87–7.83 (m, 1H), 7.80–7.75 (m, 1H), 7.68–7.64 (m, 2H), 7.51–7.45 (m, 4H), 7.41–7.37 (m, 2H), 3.94 (s, 2H), 3.11 (t, *J* = 7.2 Hz, 2H), 2.96 (t, *J* = 7.2 Hz, 2H), 2.33 (s, 3H). ^13^C-NMR *δ* (ppm): 198.4 (C=O), 135.4 (Cq), 134.4 (Cq), 133.8 (Cq), 132.3 (Cq), 131.6, 129.4, 128.3, 128.0, 127.9 (Cq), 127.3, 125.7, 125.5, 124.9, 124.6, 61.0, 52.7, 42.3, 37.0. Anal. Calcd. For C_21_H_20_BrNO: C, 65.98; H, 5.27; N, 3.66. Found: C, 66.12; H, 5.35; N, 3.73.

*1-(Benzo[d][1,3]dioxol-5-yl)-3-(methyl(naphthalen-1-ylmethyl)amino)propan-1-one* (**18c**). Following the general procedure for the formation of β-aminoketones, the reaction of *N*-methyl-1-(naphthalen-1-yl)methanamine (**13g**, 527 mg, 3.08 mmol) and 1-(benzo[*d*][1,3]dioxol-5-yl)-3-(*N*,*N*-dimethylamino)- propan-1-one hydrochloride (**17c**, 799 mg, 3.10 mmol) in a mixture of 1,4-dioxane (5.0 mL) and TEA (1.0 mL) afforded compound **18c** as a yellow solid (567 mg, 53% yield). M.p. = 91–92 °C (amorphous). FTIR (KBr): 3045, 2981, 2950, 2904, 2795, 2764, 1669 (C=O), 1601, 1503, 1256, 1036 cm^−1^. ^1^H-NMR *δ* (ppm): 8.27–8.22 (m, 1H), 7.86–7.82 (m, 1H), 7.77 (d, *J* = 6.8 Hz, 1H), 7.51–7.36 (m, 6H), 6.77 (d, *J* = 8.0 Hz, 1H), 6.03 (s, 2H, OCH_2_O), 3.95 (s, 2H), 3.11 (t, *J* = 7.2 Hz, 2H), 2.96 (t, *J* = 7.2 Hz, 2H), 2.30 (s, 3H). ^13^C-NMR *δ* (ppm): 197.6 (C=O), 151.5 (Cq), 148.0 (Cq), 134.6 (C), 133.8 (Cq), 132.4 (Cq), 131.8 (Cq), 128.3, 127.9, 127.4, 125.7, 125.5, 125.0, 124.7, 124.2, 107.8, 107.7, 101.7 (OCH_2_O), 61.0, 53.2, 42.2, 36.7. Anal. Calcd. For C_22_H_21_NO_3_: C, 76.06; H, 6.09; N, 4.03. Found: C, 76.21; H, 6.25; N, 3.86.

*3-(Methyl(naphthalen-1-ylmethyl)amino)-1-(3,4,5-trimethoxyphenyl)propan-1-one* (**18d**). Following the general procedure for the formation of β-aminoketones, the reaction of *N*-methyl-1-(naphthalen-1-yl)methanamine (**13g**, 498 mg, 2.91 mmol) and 3-(*N*,*N*-dimethylamino)-1-(3,4,5-trimethoxy- phenyl)propan-1-one hydrochloride **(17d**, 896 mg, 2.95 mmol) in a mixture of 1,4-dioxane (5.0 mL) and TEA (1.0 mL) afforded compound **18d** as a yellow solid (572 mg, 50% yield). M.p. = 94 °C (amorphous). FTIR (KBr): 3045, 2940, 2835, 2794, 1676 (C=O), 1584, 1504, 1459, 1412, 1336, 1126, 1003 cm^−1^. ^1^H NMR *δ* (ppm): 8.25–8.20 (m, 1H), 7.85–7.80 (m, 1H), 7.76 (d, *J* = 7.6 Hz, 1H), 7.76 (d, *J* = 7.6 Hz, 1H), 7.49–7.35 (m, 4H), 7.12 (s, 2H), 3.96 (s, 2H), 3.91 (s, 3H), 3.84 (s, 6H), 3.13 (t, *J* = 7.2 Hz, 2H), 2.98 (t, *J* = 7.2 Hz, 2H), 2.34 (s, 3H). ^13^C-NMR *δ* (ppm): 198.2 (C=O), 152.9 (Cq), 142.4 (Cq), 134.5 (Cq), 133.8 (Cq), 132.3 (Cq), 132.1 (Cq), 128.3, 127.9, 127.3, 125.7, 125.5, 125.0, 124.6, 105.4, 61.1, 60.8 (OCH_3_), 56.1 (OCH_3_), 53.2, 42.4, 36.9. Anal. Calcd. For C_24_H_27_NO_4_: C, 73.26; H, 6.92; N, 3.56. Found: C, 73.41; H, 6.87; N, 3.67.

*(±)-3-(N-Methyl-N-((naphthalen-5-yl)methyl)amino)-1-phenylpropan-1-ol* (**19a**). Following the general procedure for the formation of γ-aminoalcohols, the reaction of β-aminoketone **18a** (394 mg, 1.30 mmol) and NaBH_4_ (98 mg, 2.60 mmol) in 5.0 mL of MeOH afforded compound **19a** as a yellow solid. Yield: 93% (369 mg). M.p. = 75–76 °C (lit. [18], 76–77 °C).

*(±)-1-(4-Bromophenyl)-3-(methyl(naphthalen-1-ylmethyl)amino)propan-1-ol* (**19b**). Following the general procedure for the formation of γ-aminoalcohols, the reaction of β-aminoketone **18b** (510 mg, 1.34 mmol) and NaBH_4_ (101 mg, 2.68 mmol) in 5.0 mL of MeOH afforded compound **19b** as a yellow oil. Yield: 83% (427 mg). FTIR (film): 3374, 3046, 2948, 2801, 1593, 1509, 1485, 1463, 1072, 1048, 1009 cm^−1^. ^1^H-NMR *δ* (ppm): 8.18 (d, *J* = 8.8 Hz, 1H), 7.91 (d, *J* = 8.0 Hz, 1H), 7.85 (d, *J* = 8.0 Hz, 1H), 7.63–7.58 (m, 1H), 7.56–7.51 (m, 1H), 7.46–7.38 (m, 2H), 7.31 (d, *J* = 8.4 Hz, 2H), 6.95 (d, *J* = 8.4 Hz, 2H), 6.19 (br s, 1H, OH), 4.74 (dd, *J* = 3.2, 7.6 Hz, 1H), 4.03 (d, *J* = 12.8 Hz, 1H), 3.86 (d, *J* = 12.8 Hz, 1H), 2.80–2.72 (m, 1H), 2.69–2.62 (m, 1H), 2.40 (s, 3H), 1.96–1.88 (m, 1H), 1.86–1.75 (m, 1H). ^13^C-NMR *δ* (ppm): 143.8 (Cq), 133.9 (Cq), 133.3 (Cq), 132.3 (Cq), 131.0, 128.6, 128.4, 128.1, 127.2, 126.3, 125.9, 125.1, 123.9, 120.3 (Cq), 74.5, 61.2, 55.6, 42.2, 34.3. Anal. Calcd. For C_21_H_22_BrNO: C, 65.63; H, 5.77; N, 3.64. Found: C, 65.70; H, 5.86; N, 3.58.

*(±)-1-(Benzo[d][1,3]dioxol-5-yl)-3-(methyl(naphthalen-1-ylmethyl)amino)propan-1-ol* (**19c**). Following the general procedure for the formation of γ-aminoalcohols, the reaction of β-aminoketone **18c** (495 mg, 1.42 mmol) and NaBH_4_ (108 mg, 2.85 mmol) in 5.0 mL of MeOH afforded compound **19c** as a white solid. Yield: 93% (461 mg). M.p. = 96–98 °C. FT-IR (KBr): 3373, 3045, 2948, 2886, 2841, 2800, 1599, 1503, 1486, 1441, 1241, 1039 cm^−1^. ^1^H-NMR *δ* (ppm): 8.21 (d, *J* = 8.4 Hz, 1H), 7.90 (d, *J* = 8.0 Hz, 1H), 7.86–7.81 (m, 1H), 7.64–7.58 (m, 1H), 7.55–7.50 (m, 1H), 7.47–7.41 (m, 2H), 6.74 (d, *J* = 1.2 Hz, 1H), 6.68 (d, *J* = 8.0 Hz, 1H), 6.61 (dd, *J* = 1.2, 7.8 Hz, 1H), 5.98 (br s, 1H, OH), 5.91 (s, 2H), 4.72 (dd, *J* = 5.6, 5.6 Hz, 1H), 3.99 (d, *J* = 12.8 Hz, 1H), 3.93 (d, *J* = 12.8 Hz, 1H), 2.86–2.78 (m, 1H), 2.70–2.63 (m, 1H), 2.37 (s, 3H), 1.90–1.84 (m, 2H). ^13^C-NMR *δ* (ppm): 147.4 (Cq), 147.4 (Cq), 146.2 (Cq), 139.0 (Cq), 133.9 (Cq), 133.4 (Cq), 132.3 (Cq), 128.6, 128.4, 127.9, 126.3, 125.8, 125.1, 123.9, 118.6, 107.8, 106.2, 100.7, 75.1, 61.1, 56.2, 42.1, 34.8. Anal. Calcd. For C_22_H_23_NO_3_: C, 75.62; H, 6.63; N, 4.01. Found: C, 75.70; H, 6.75; N, 4.15.

*(±)-3-(Methyl(naphthalen-1-ylmethyl)amino)-1-(3,4,5-trimethoxyphenyl)propan-1-ol* (**19d**). Following the general procedure for the formation of γ-aminoalcohols, the reaction of β-aminoketone **18d** (520 mg, 1.32 mmol) and NaBH_4_ (100 mg, 2.64 mmol) in 5.0 mL of MeOH afforded compound **19d** as a yellow oil. Yield: 90% (470 mg). FT-IR (film): 3414, 3050, 2940, 2834, 1592, 1506, 1461, 1417, 1232, 1182, 1126, 1009 cm^−1^. ^1^H-NMR *δ* (ppm): 8.22 (d, *J* = 8.4 Hz, 1H), 7.88 (d, *J* = 8.0 Hz, 1H), 7.82 (dd, *J* = 1.8, 7.0 Hz, 1H), 7.61–7.56 (m, 1H), 7.54–7.42 (m, 3H), 6.56 (s, 2H), 6.07 (br s, 1H, OH), 4.72 (dd, *J* = 2.4, 9.0 Hz, 1H), 4.01 (d, *J* = 13.0 Hz, 1H), 3.96 (d, *J* = 13.0 Hz, 1H), 3.83 (s, 9H), 2.99–2.90 (m, 1H), 2.74–2.65 (m, 1H), 2.36 (s, 3H), 2.01–1.88 (m, 1H), 1.87–1.79 (m, 1H). ^13^C-NMR *δ* (ppm): 153.0 (Cq), 140.7 (Cq), 136.7 (Cq), 133.8 (Cq), 133.4 (Cq), 132.2 (Cq), 128.6, 128.30, 127.8, 126.2, 125.7, 125.1, 123.8, 102.4, 75.5, 61.0, 60.7, 56.9, 56.0, 43.0, 35.0. Anal. Calcd. For C_24_H_29_NO_4_: C, 72.89; H, 7.39; N, 3.54. Found: C, 72.95; H, 7.50; N, 3.42.

*(±)-1,1′-(1,4-Phenylene)bis(3-(methyl(naphthalen-1-ylmethyl)amino)propan-1-ol* (**19e**). Following the general procedure for the formation of γ-aminoalcohols, the reaction of β-aminoketone **18e** (481 mg, 0.91 mmol) and NaBH_4_ (69 mg, 1.82 mmol) in 5.0 mL of MeOH afforded compound **19e** as a yellow oil. Yield: 80% (388 mg). FT-IR (film): 3363, 3048, 2948, 2841, 2800, 1463, 1129, 1076, 1049, 1021 cm^−1^. ^1^H-NMR *δ* (ppm): 8.21 (d, *J* = 8.0 Hz, 2H), 7.88 (d, *J* = 8.0 Hz, 2H), 7.85–7.79 (m, 2H), 7.63–7.56 (m, 2H), 7.54–7.48 (m, 2H), 7.46–7.39 (m, 4H), 7.06 (s, 2H), 7.05 (s, 2H), 5.89 (br s, 2H, OH), 4.80–4.74 (m, 2H), 3.99 (d, *J* = 13.1 Hz, 1H), 3.99 (d, *J* = 12.8 Hz, 1H), 3.92 (d, *J* = 13.1 Hz, 1H), 3.92 (d, *J* = 12.8 Hz, 1H), 2.85–2.76 (m, 2H), 2.69–2.61 (m, 2H), 2.36 (s, 6H), 1.93–1.85 (m, 4H). ^13^C-NMR *δ* (ppm): 143.3 (Cq), 133.9 (Cq), 133.5 (Cq), 132.3 (Cq), 128.6, 128.3, 127.9, 126.3, 125.8, 125.2, 125.1, 124.0, 75.0, 61.1, 56.3, 42.1, 34.7. Anal. Calcd. For C_36_H_40_N_2_O_2_: C, 81.17; H, 7.57; N, 5.26. Found: C, 81.26; H, 7.65; N, 5.01.

*(±)-3-Benzyl(2-hydroxyethyl)amino)-1-phenylpropan-1-ol* (**20a**). Following the general procedure for the formation of γ-aminoalcohols, the reaction of β-aminoketone **23a** (521 mg, 1.84 mmol) and NaBH_4_ (139 mg, 3.68 mmol) in 5.0 mL of MeOH afforded compound **20a** as a yellow oil. Yield: 94% (494 mg). FT-IR (film): 3396, 2943, 2827, 1603, 1129, 1059, 1031 cm^−1^. ^1^H-NMR *δ* (ppm): 7.39–7.22 (m, 10H), 4.84 (dd, *J* = 3.5, 8.8 Hz, 1H), 3.81 (d, *J* = 13.3 Hz, 1H), 3.75–3.64 (m, 2H), 3.55 (d, *J* = 13.1 Hz, 1H), 2.86 (ddd, *J* = 4.5, 8.8, 13.2 Hz, 1H), 2.79–2.69 (m, 3H), 2.61 (ddd, *J* = 4.4, 5.7, 13.3 Hz, 1H), 1.89–1.82 (m, 2H), OH is absent. ^13^C-NMR *δ* (ppm): 144.6 (Cq), 137.9 (Cq), 129.3, 128.6, 128.3, 127.5, 127.2, 125.6, 74.9, 59.9, 59.6, 56.2, 52.8, 35.3. Anal. Calcd. For C_18_H_23_NO_2_: C, 72.76; H, 8.12; N, 4.91. Found: C, 72.85; H, 8.01; N, 5.06.

*(±)-2-Benzyl(2-hydroxyethyl)amino)-1-(4-methoxyphenyl)propan-1-ol* (**20f**). Following the general procedure for the formation of γ-aminoalcohols, the reaction of β-aminoketone **23f** (530 mg, 1.69 mmol) and NaBH_4_ (128 mg, 3.38 mmol) in 5.0 mL of MeOH afforded compound **20f** as a yellow solid. Yield: 87% (464 mg). M.p. = 68–69 °C. FT-IR (KBr): 3375, 2949, 2835, 1611, 1176, 1130, 1034 cm^−1^. ^1^H-NMR *δ* (ppm): 7.37–7.29 (m, 5H), 7.23 (d, *J* = 8.8 Hz, 2H), 6.85 (d, *J* = 8.5 Hz, 2H), 5.52 (br s, 1H, OH), 4.78 (dd, *J* = 3.3, 8.6 Hz, 1H), 3.80 (s, 3H), 3.80 (d, *J* = 13.1 Hz, 1H), 3.73–3.63 (m, 2H), 3.54 (d, *J* = 13.1 Hz, 1H), 2.87–2.67 (m, 4H), 2.59 (ddd, *J* = 5.1, 5.1, 13.1 Hz, 1H), 1.98–1.88 (m, 1H), 1.77–1.85 (m, 1H). ^13^C-NMR *δ* (ppm): 158.7 (Cq), 137.9 (Cq), 136.8 (Cq), 129.2, 128.5, 127.4, 126.7, 113.6, 74.4, 59.8, 59.5, 56.1, 55.2, 52.7, 35.2. Anal. Calcd. For C_19_H_25_NO_3_: C, 72.35; H, 7.99; N, 4.44. Found: C, 72.51; H, 8.10; N, 4.60.

#### 3.2.5. General Procedure for the Synthesis of Allylamines **21** and **22**

A mixture of the γ-aminoalcohol **19** and **20** (300 mg) and 5N HCl solution (5 mL) was stirred at reflux during 2–3 h. After reaction finished (TLC control), the mixture was neutralized with 5N NaOH until pH = 8. Then, solution was extracted with EtOAc (3 × 5 mL), the combined organic layers were dried with anhydrous Na_2_SO_4_ and the solvent was removed under reduced pressure. Crudes were purified by column chromatography on silica gel, using a mixture of CHCl_3_:MeOH (40:1) as eluent.

*(E)-N-Methyl-N-(naphthalen-1-ylmethyl)-3-phenylprop-2-en-1-amine* (**21a**)*, naftifine*. Following the strategy 2 for the formation of allylamines, the reaction of γ-aminoalcohol **19a** (310 mg, 1.01 mmol) and 5N HCl solution (5.0 mL) afforded compound **21a** as a yellow oil (lit. [10] 94%, lit. [11] 82%, lit. [12], 68%). Yield: 90% (310 mg). FT-IR (film): 3028, 2943, 2835, 2786, 1596, 1509, 1451, 1362, 1127, 1013 cm^−1^. ^1^H-NMR *δ* (ppm): 8.36 (d, *J* = 8.4 Hz, 1H), 7.90 (d, *J* = 7.6 Hz, 1H), 7.83 (d, *J* = 8.0 Hz, 1H), 7.62–7.43 (m, 6H), 7.37 (t, *J* = 7.2 Hz, 2H), 7.32–7.25 (m, 1H), 6.63 (d, *J* = 15.6 Hz, 1H), 6.43 (td, *J* = 6.4, 15.6 Hz, 1H), 4.01 (s, 2H), 3.34 (d, *J* = 6.0 Hz, 2H), 2.34 (s, 3H). ^13^C-NMR *δ* (ppm): 137.1 (Cq), 134.8 (Cq), 133.9 (Cq), 132.7, 132.5 (Cq), 128.5, 128.4, 127.9, 127.5, 127.4, 127.3, 126.3, 125.8, 125.5, 125.1, 124.6, 60.3, 60.1, 42.4. Anal. Calcd. For C_21_H_21_N: C, 87.76; H, 7.37; N, 4.87. Found: C, 87.83; H, 7.45; N, 4.95.

*(E)-3-(4-Bromophenyl)-N-methyl-N-(naphthalen-1-ylmethyl)prop-2-en-1-amine* (**21b**). Following the strategy 2 for the formation of allylamines, the reaction of γ-aminoalcohol **19b** (301 mg, 0.78 mmol) and 5N HCl solution (5.0 mL) afforded compound **21b** as a yellow solid. Yield: 81% (231 mg). M.p. = 55–57 °C (lit. [35], 84%).

*(E)-3-(Benzo[d][1,3]dioxol-5-yl)-N-methyl-N-(naphthalen-1-ylmethyl)prop-2-en-1-amine* (**21c**). Following the strategy 2 for the formation of allylamines, the reaction of γ-aminoalcohol **19c** (325 mg, 0.93 mmol) and 5N HCl solution (5.0 mL) afforded compound **21c** as a yellow oil. Yield: 90% (277 mg). FT-IR (film): 3040, 2979, 2943, 2885, 2835, 2783, 1600, 1487, 1443, 1249, 1039 cm^−1^. ^1^H-NMR *δ* (ppm): 8.33 (d, *J* = 8.0 Hz, 1H), 7.88 (d, *J* = 7.6 Hz, 1H), 7.80 (d, *J* = 7.6 Hz, 1H), 7.58–7.41 (m, 4H), 6.98 (d, *J* = 1.2 Hz, 1H), 6.84 (dd, *J* = 1.2, 8.0 Hz, 1H), 6.78 (d, *J* = 8.0 Hz, 1H), 6.51 (d, *J* = 16.0 Hz, 1H), 6.22 (td, *J* = 6.8, 15.6 Hz, 1H), 5.98 (s, 2H, OCH_2_O), 3.96 (s, 2H), 3.28 (d, *J* = 6.0 Hz, 2H), 2.30 (s, 3H). ^13^C-NMR *δ* (ppm): 148.0 (Cq), 147.0 (Cq), 134.9 (Cq), 133.9 (Cq), 132.5 (Cq), 132.2, 131.6 (Cq), 128.4, 127.9, 127.4, 125.8, 125.7, 125.5, 125.1, 124.6, 120.8, 108.2, 105.7, 101.0, 60.3, 60.0, 42.4. Anal. Calcd. For C_22_H_21_NO_2_: C, 79.73; H, 6.39; N, 4.23. Found: C, 79.90; H, 6.45; N, 4.05.

*(E)-N-Methyl-N-(naphthalen-1-ylmethyl)-3-(3,4,5-trimethoxyphenyl)prop-2-en-1-amine* (**21d**). Following the strategy 2 for the formation of allylamines, the reaction of γ-aminoalcohol **19d** (314 mg, 0.79 mmol) and 5N HCl solution (5.0 mL) afforded compound **21d** as a yellow oil. Yield: 70% (209 mg). FT-IR (film): 3041, 2938, 2834, 2786, 1581, 1505, 1457, 1416, 1331, 1239, 1126, 1011 cm^−1^. ^1^H-NMR *δ* (ppm): 8.33 (d, *J* = 8.4 Hz, 1H), 7.88 (d, *J* = 7.6 Hz, 1H), 7.80 (d, *J* = 7.6 Hz, 1H), 7.59–7.41 (m, 4H), 6.64 (s, 2H), 6.52 (d, *J* = 15.6 Hz, 1H), 6.29 (td, *J* = 6.8, 15.6 Hz, 1H), 3.98 (s, 2H), 3.89 (s, 6H), 3.87 (s, 3H), 3.30 (d, *J* = 6.4 Hz, 2H), 2.32 (s, 3H). ^13^C-NMR *δ* (ppm): 153.2 (Cq), 137.6 (Cq), 134.7 (Cq), 133.8 (Cq), 132.8 (Cq), 132.4 (Cq), 132.4, 128.4, 127.9, 127.4, 127.2, 125.8, 125.5, 125.1, 124.5, 103.3, 60.8, 60.3, 60.2, 56.0, 42.5. Anal. Calcd. For C_24_H_27_NO_3_: C, 76.36; H, 7.21; N, 3.71. Found: C, 76.45; H, 7.10; N, 3.86.

*(E)-N-Methyl-3-(4-((E)-3-(methyl(naphthalen-1-ylmethyl)amino)prop-1-en-1-yl)phenyl)-N-(naphthalen-2-ylmethyl)prop-2-en-1-amine* (**21e**). Following the strategy 2 for the formation of allylamines, the reaction of γ-aminoalcohol **19e** (295 mg, 0.55 mmol) and 5N HCl solution (5.0 mL) afforded compound **21e** as a yellow oil. Yield: 78% (213 mg). FT-IR (film): 3042, 2943, 2834, 2785, 1596, 1509, 1456, 1013 cm^−1^. ^1^H-NMR *δ* (ppm): 8.36 (d, *J* = 8.4 Hz, 2H), 7.90 (d, *J* = 8.4 Hz, 2H), 7.83 (d, *J* = 8.0 Hz, 2H), 7.61–7.44 (m, 8H), 7.39 (s, 4H), 6.61 (d, *J* = 16.0 Hz, 2H), 6.42 (td, *J* = 6.8, 16.0 Hz, 2H), 3.33 (d, *J* = 6.8 Hz, 4H), 4.00 (s, 4H), 2.33 (s, 6H). ^13^C-NMR *δ* (ppm): 136.2 (Cq), 134.8 (Cq), 133.9 (Cq), 132.5 (Cq), 132.3, 128.4, 127.9, 127.4 (× 2C), 126.5, 125.8, 125.5, 125.1, 124.6, 60.4, 60.1, 42.4. Anal. Calcd. For C_36_H_36_N_2_: C, 87.05; H, 7.31; N, 5.64. Found: C, 87.22; H, 7.44; N, 5.45.

*(E)-2-(Benzyl(cinnamyl)amino)ethan-1-ol* (**22a**). Following the strategy 2 for the formation of allylamines, the reaction of γ-aminoalcohol **20a** (331 mg, 1.16 mmol) and 5N HCl solution (5.0 mL) afforded compound **22a** as a yellow oil. Yield: 70% (217 mg). FT-IR (film): 3424, 2944, 2880, 1599, 1580, 1127, 1053, 1028 cm^−1^. ^1^H-NMR *δ* (ppm): 7.42–7.25 (m, 10H), 6.55 (d, *J* = 16.0 Hz, 1H), 6.29 (td, *J* = 6.8, 16.0 Hz, 1H), 3.73 (s, 2H), 3.65 (t, *J* = 5.4 Hz, 2H), 3.34 (dd, *J* = 1.0, 6.8 Hz, 2H), 2.76 (t, *J* = 5.4 Hz, 2H), 2.43 (br s, 1H, OH). ^13^C-NMR *δ* (ppm): 138.7 (Cq), 136.8 (Cq), 133.2, 129.0, 128.5, 128.4, 127.5, 127.2, 126.5, 126.3, 58.6, 58.2, 56.0, 54.8. Anal. Calcd. For C_18_H_21_NO: C, 80.86; H, 7.92; N, 5.24. Found: C, 80.97; H, 8.04; N, 5.33.

*(E)-2-(Benzyl(3-(4-methoxyphenyl)allyl)amino)ethan-1-ol* (**22f**). Following the strategy 2 for the formation of allylamines, the reaction of γ-aminoalcohol **20f** (319 mg, 1.01 mmol) and 5N HCl solution (5.0 mL) afforded compound **22f** as a colorless oil. Yield: 68% (204 mg) (lit. [36], yellow oil).

### 3.3. Antifungal Evaluation

#### 3.3.1. Microorganisms and Media

For the antifungal evaluation, standardized strains from the American Type Culture Collection (ATCC), Rockville, MD, USA, and CEREMIC (CCC), Centro de Referencia en Micología, Facultad de Ciencias Bioquímicas y Farmacéuticas, Suipacha 531-(2000)-Rosario, Argentina, were used: *C. albicans* ATCC 10231, *S. cerevisiae* ATCC 9763, *C. neoformans* ATCC 32264, *Aspergillus flavus* ATCC 9170, *Aspergillus fumigatus* ATTC 26934, *Aspergillus niger* ATCC 9029, *Trichophyton rubrum* CCC 113, *Trichophyton mentagrophytes* ATCC 9972, and *Microsporum gypseum* CCC 115. Strains were grown on Sabouraud-chloramphenicol agar slants for 48 h at 30 °C, maintained on slopes of Sabouraud-dextrose agar (SDA, Oxoid, Cambridge, UK), and subcultured every 15 days to prevent pleomorphic transformations. Inocula of cell or spore suspensions were obtained according to reported procedures [26,27] and adjusted to 1–5 × 10^3^ cells/spores with colony forming units (CFU)/mL.

#### 3.3.2. Antifungal Susceptibility Testing

Minimum inhibitory concentration (MIC) of each compound was determined by using broth microdilution techniques according to the guidelines of the Clinical and Laboratory Standards Institute for yeasts (M27-A3) [26] and for filamentous fungi (including dermatophytes) (M38-A2) [27]. MIC values were determined in RPMI-1640 (Sigma-Aldrich) buffered to pH 7.0 with MOPS. Microtiter trays were incubated at 35 °C for yeasts and *Aspergillus* spp. and at 28–30 °C for dermatophyte strains in a moist, dark chamber, and MICs were visually recorded at 48 h for yeasts, and at a time according to the control fungus growth, for the rest of fungi. For the assay, stock solutions of pure compounds were two-fold diluted with RPMI from 250 to 1.0 µg/mL (=250, 125, 62.5, 31.3, 15.6, 7.8, 3.9, 2.0, 1.0 and 0.5 µg/mL) (final volume = 100 µL) and a final DMSO concentration ≤1%. A volume of 100 µL of inoculum suspension was added to each well with the exception of the sterility control where sterile water was added to the well instead. Terbinafine (obtained from the commercial drug Lamisil from Novartis Co., Basel, Switzerland) and amphotericin B were used as positive controls. Endpoints were defined as the lowest concentration of drug resulting in total inhibition (MIC_100_) of visual growth compared to the growth in the control wells containing no antifungal drug. In addition to MIC determinations, the evaluation of Minimum Fungicide Concentration (MFC) of each compound against the fungal panel was accomplished by subculturing a sample of media from MIC tubes showing no growth, onto drug-free agar plates.

#### 3.3.3. Fungal Growth Inhibition Percentage Determination

Yeasts broth microdilution technique M27-A3 of CLSI [26] was performed in 96-well microplates. For the assay, compound test wells (CTWs) were prepared with stock solutions of each compound in DMSO (maximum concentration ≤1%), diluted with RPMI-1640, to final concentrations of 250–3.9 μg/mL^−1^. An inoculum suspension (100 μL) was added to each well (final volume in the well = 200 μL). A growth control well (GCW) (containing medium, inoculum, and the same amount of DMSO used in a CTW, but compound-free) and sterility control well (SCW) (sample, medium, and sterile water instead of inoculum) were included for each fungus tested. Microtiter trays were incubated in a moist, dark chamber at 30 °C for 48 h for both yeasts. Microplates were read in a VERSA Max microplate reader (Molecular Devices, Sunnyvale, CA, USA). Amphotericin B was used as positive control. Tests were performed in triplicate. Reduction of growth for each compound concentration was calculated as follows: % of inhibition = 100 − (OD_405_ CTW − OD_405_ SCW)/(OD_405_ GCW − OD_405_ SCW). The means ± SD (standard deviations) were used for constructing the dose-response curves representing % inhibition *vs.* concentration of each compound. Dose-response curves were constructed with SigmaPlot 11.0 software.

#### 3.3.4. MIC_100_, MIC_80_ and MIC_50_ Determinations

Three endpoints were defined from the dose-response curves. Minimum Inhibitory concentration (MIC) resulting in total fungal growth inhibition was named MIC_100_, while MIC_80_ and MIC_50_ were defined as the minimum concentration that inhibits 80% or 50% of the fungal growth, respectively.

## 4. Conclusions

In summary, we have developed two efficient and straightforward approaches for the synthesis of naftifine and diversely substituted analogues **16** and **20** mediated by a Mannich-type reaction in at least one step of each approach. Strategy 1 involved a two-step (both Mannich- and aza-Prins- combined reactions) sequence, mediated by an uncatalyzed three-component Mannich-type reaction leading to γ-aminoalcohols **15** as the key intermediates for allylamines **16**. Particularly, we were able to obtain naftifine in a one-pot fashion through Strategy 1 starting from styrene. Although naftifine was isolated in a relatively lower yield than in previous approaches, remarkably, this strategy represents the first direct method for the synthesis of this antifungal compound. Strategy 2 consisted in a three-step sequence involving a one-pot synthesis of the γ-aminoalcohols **19** and **20** as the key intermediates for allylamines **21** and **22**. In general, naftifine and the target products **16**, **21** and **22** were obtained in good to excellent yields after their corresponding dehydration processes catalyzed whether by Brønsted or Lewis acids like H_2_SO_4_, HCl and AlCl_3_, respectively. The synthesized compounds were tested for antifungal properties against a panel of clinically important fungi. Most compounds were inactive against *Aspergillus* spp., while showed relevant activities against the dermatophytes *T. rubrum* and *T. mentagrophytes*. The most active compounds **18b** and **18c** possessed a β-aminoketo structure, and among them, compounds **18b**, **18c** and **21c** were 4-Br (**18b**) or 3,4-methylenedioxy (**18c** and **21c**) substituted in their R^1^ groups. Interesting enough, **18b** displayed high activities also against yeasts, with a MIC_80_ against *C. neoformans* and *C. albicans* of 7.8 µg/mL. This is a relevant low value that positions **18b** as a good hit candidate for future research. In addition, **18b** is fungicide rather than fungistatic with MFC value of 15.6 µg/mL against *C. neoformans*.

## Figures and Tables

**Table 1:** MIC values (µg/mL) of allylamine derivatives **18**, **19** and **21** acting against human opportunistic pathogenic fungi. MIC/MFC values are recorded in µg/mL.

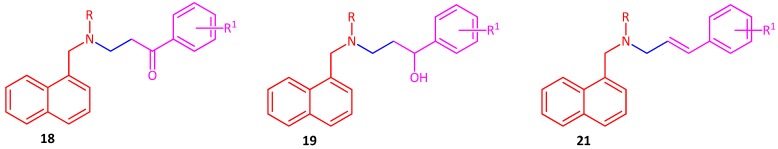												
Compound		R	R^1^	*Ca*	*Sc*	*Cn*	*Afu*	*Afl*	*An*	*Mg*	*Tr*	*Tm*
**18**	**b**	CH_3_	4-Br	15.6/62.5	15.6/31.3	7.8/15.6	7.8/15.6	250/250	250/>250	>250/>250	3.9/3.9	2.0/2.0
	**c**	CH_3_	3,4-OCH_2_O	125/125	125/125	31.25/62.5	31.3/62.5	250/250	250/>250	>250/>250	7.8/15.6	7.8/15.6
**19**	**b**	CH_3_	4-Br	125/250	125/250	62.5/125	250/>250	250/>250	250/>250	62.5/125	31.2/62.5	31.2/62.5
	**c**	CH_3_	3,4-OCH_2_O	>250	>250	>250	>250	>250	>250	250/250	250/250	250/250
**21**	**b**	CH_3_	4-Br	250	>250	>250	>250	>250	>250	125/>250	125/>250	125/>250
	**c**	CH_3_	3,4-OCH_2_O	125/>250	250/250	125/250	250/>250	250/>250	250/>250	125/>250	1.0/1.8	0.5/1.0
Amphotericin B				1.0/1.0	1.0/1.0	1.0/2.0	2.0/2.0	2.0/2.0	2.0/2.0	0.5/0.5	0.5/0.5	0.5/0.5
Terbinafine				-	-	-	-	-	-	0.008/0.015	0.004/0.008	0.004/0.015

*Ca*: *Candida albicans* ATCC 10231, *Sc: Saccharomyces cerevisiae* ATCC 9763, *Cn*: *Cryptococcus neoformans* ATCC 32264, *An*: *Aspergillus niger* ATCC 9029, *Afl*: *Aspergillus flavus* ATCC 9170, *Afu*: *Aspergillus fumigatus* ATCC 26934, *Mg*: *Microsporum gypseum* CCC 115, *Tr*: *Trichophyton rubrum* CCC 113, *Tm*: *Trichophyton mentagrophytes* ATCC 9972.

**Table 2:** The inhibition percentages values and Minimum Inhibitory Concentrations (MIC_100_, MIC_80_ and MIC_50_) and Minimum Fungicidal Concentration (MFC) of the naftifine-analogues **18b**, **19b** and **21b** against *C. albicans* (*Ca*) and *C. neoformans* (*Cn*).

Compound		Fungus	Concentrations of the Compounds (µg/mL)							MIC (µg/mL)			MFC (µg/mL)
			250	125	62.5	31.2	15.6	7.8	3.9	MIC_100_	MIC_80_	MIC_50_	MFC
**18b**	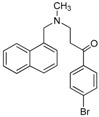	*Ca*	100	100	100	100	100	87.4 ± 1.7	28.8 ± 2.6	15.6	7.8	7.8	62.5
		*Cn*	100	100	100	100	100	98.9 ± 3.6	39.3 ± 3.7	7.8	7.8	7.8	15.6
**19b**	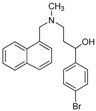	*Ca*	100	100	86.8 ± 1.3	54.3 ± 2.2	20.7 ± 3.2	7.7 ± 1.6	4.5 ± 0.7	125	62.5	31.2	250
		*Cn*	100	100	100	87.3 ± 1.6	20.2 ± 1.3	8.37 ± 1.3	0	62.5	31.2	31.2	125
**21b**	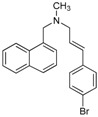	*Ca*	95.4 ± 8.3	83.2 ± 10.2	74.9 ± 5.7	52.4 ± 2.1	14.9 ± 1.6	5.6 ± 1.2	1.9 ± 1.5	250	62.5	31.2	>250
		*Cn*	58.1 ± 2.7	52.5 ± 4.2	49.4 ± 2.3	48.8 ± 1.8	35.1 ± 0.0	19.3 ± 1.2	0	>250	>250	31.2	>250
Amphotericin B		*Ca*	100	100	100	100	100	100	100	1.0	0.5	0.2	1.0
		*Cn*	100	100	100	100	100	100	100	1.2	0.5	0.2	1.2

## Supplementary Materials

The supplementary materials are available online. Copies of ^1^H- and ^13^C-NMR spectra for allylamines **16**, **21** and **22** and naftifine are available online.
