# Supplementary material for: Design of Two Alternative Routes for the Synthesis of Naftifine and Analogues as Potential Antifungal Agents
Source: Molecules. 2018 Feb 26;23(3):520. doi: 10.3390/molecules23030520 (PMC6017661; doi:10.3390/molecules23030520)

Supplementary Material for: Design of two alternative routes for the synthesis of naftifine and analogues as potential antifungal agents

Rodrigo Abonia, Alexander Garay, Juan C. Castillo, Braulio Insuasty, Jairo Quiroga, Manuel Nogueras, Justo Cobo, Estefanía Butassi and Susana Sacchino

Content

- Copies of ^{1}H, ^{13}C and DEPT-135 NMR spectra for allylamines **16**, **21-22** and naftifine

Compound **16a**

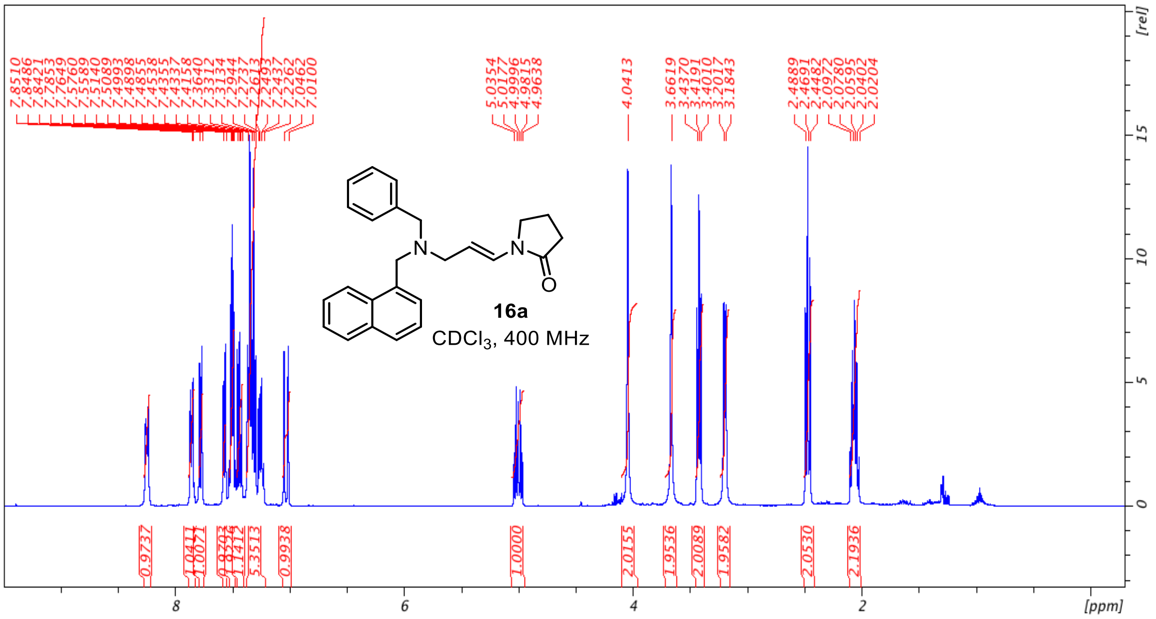

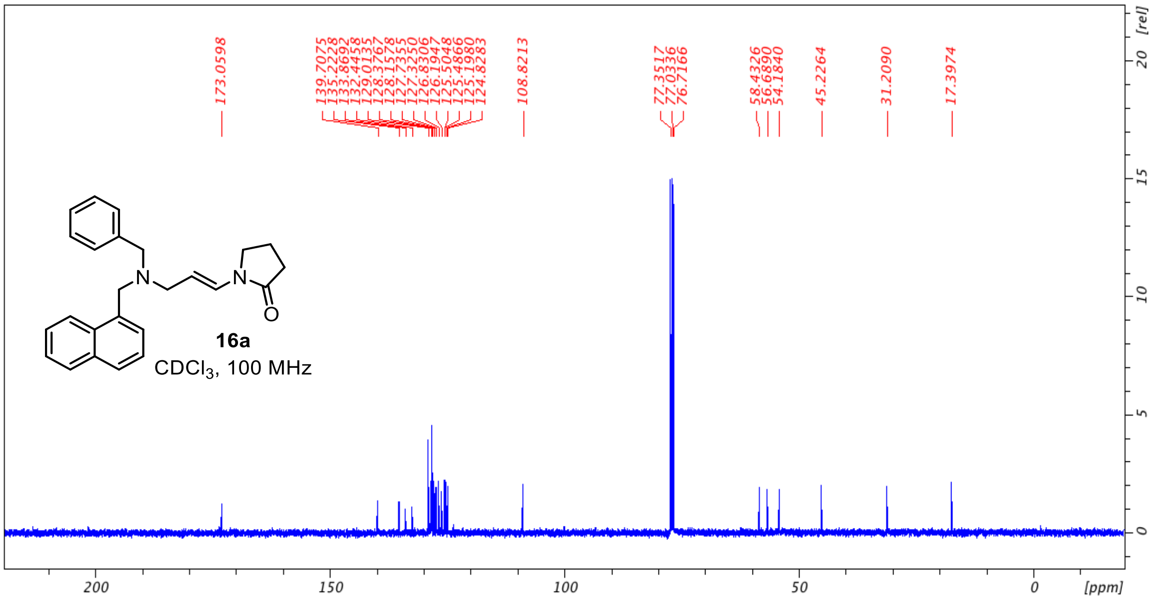

Compound **16b**

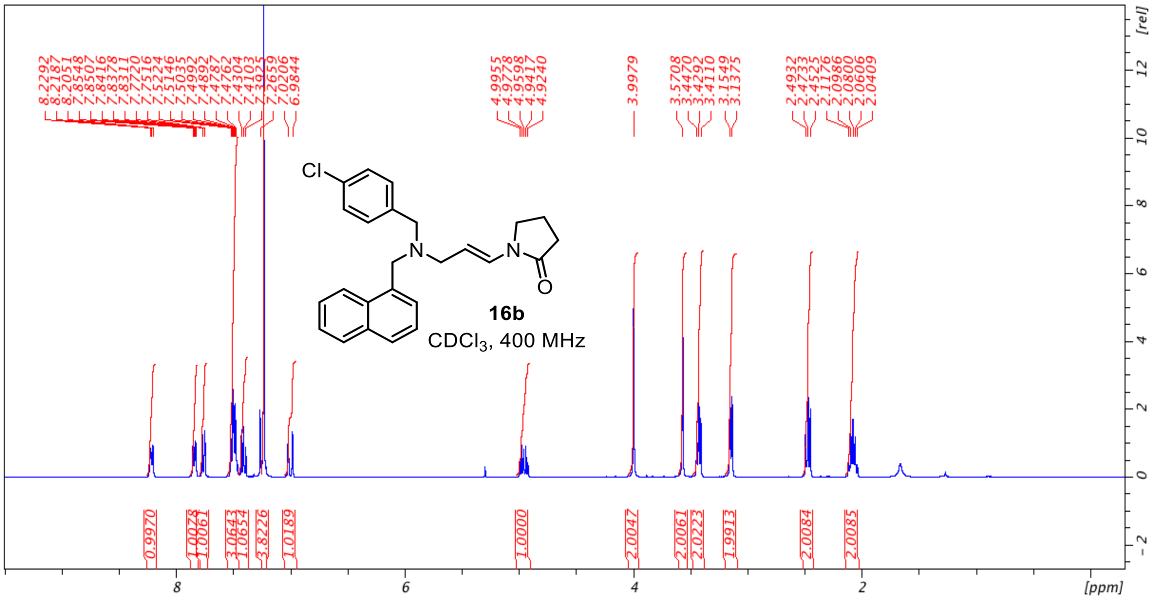

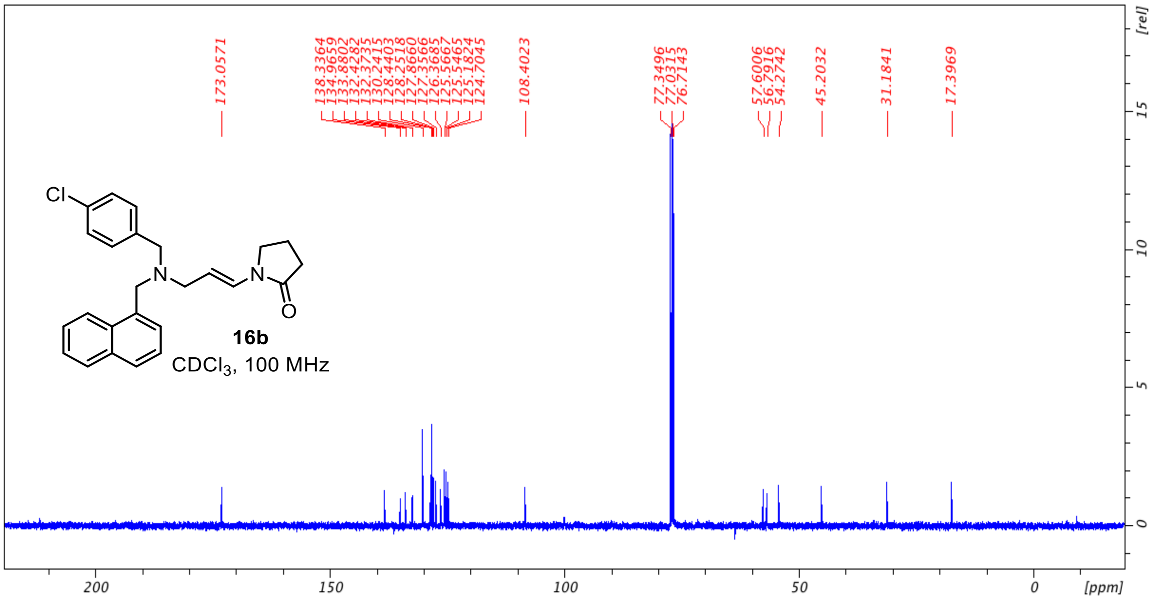

Compound **16c**

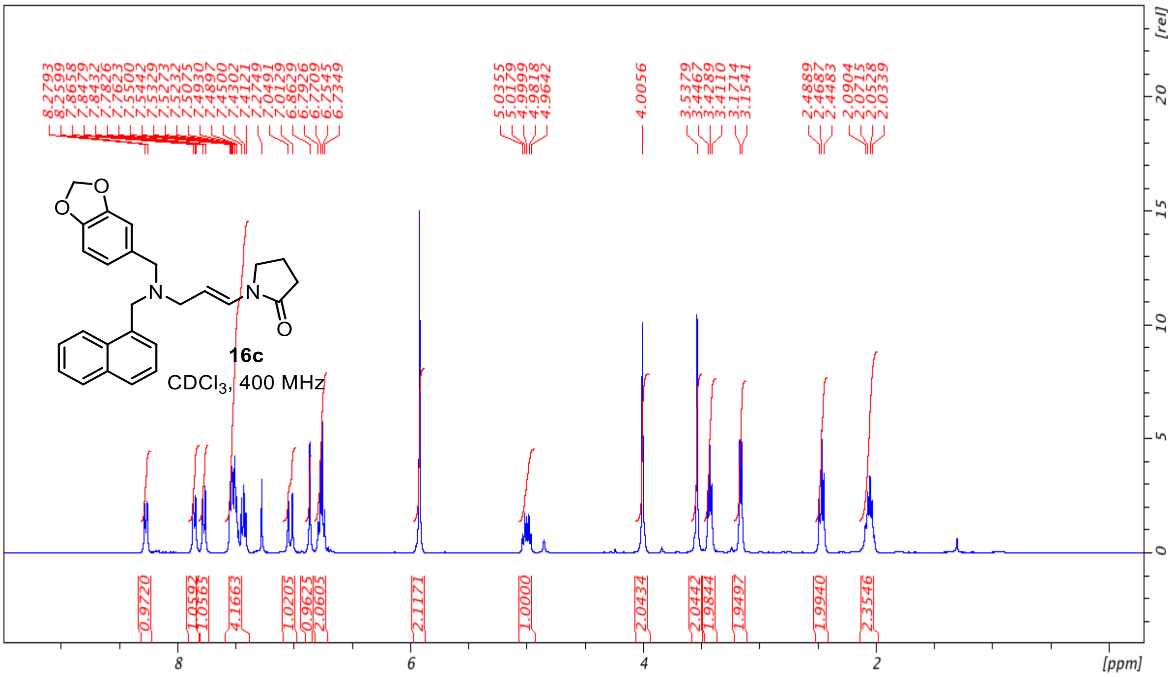

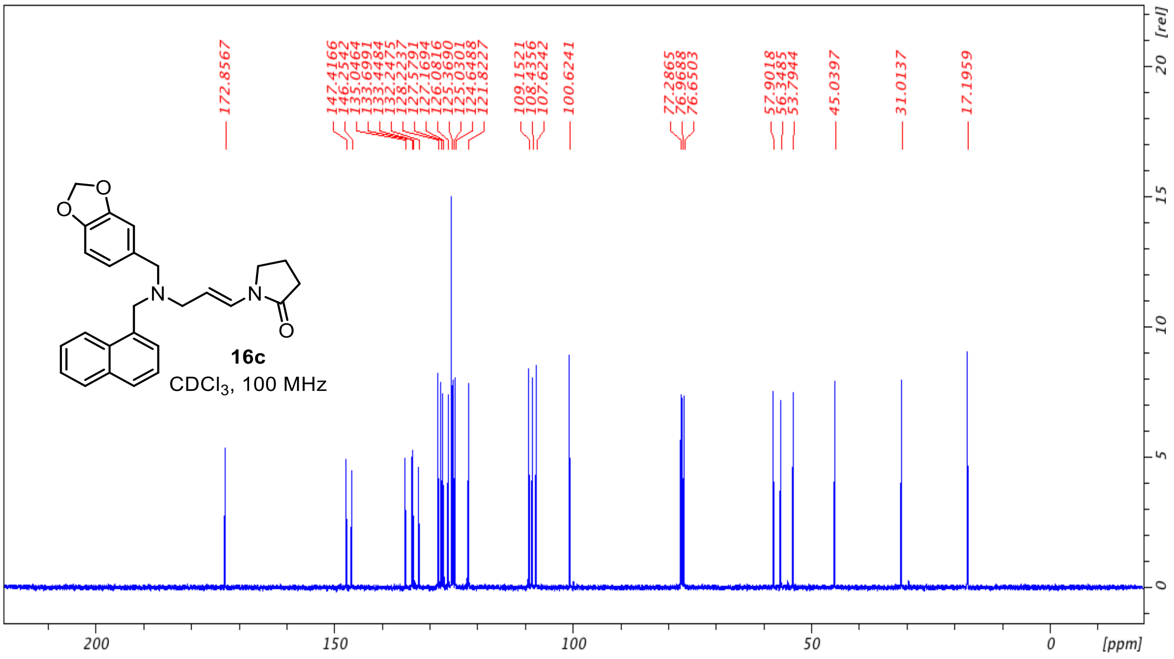

Compound **16d**

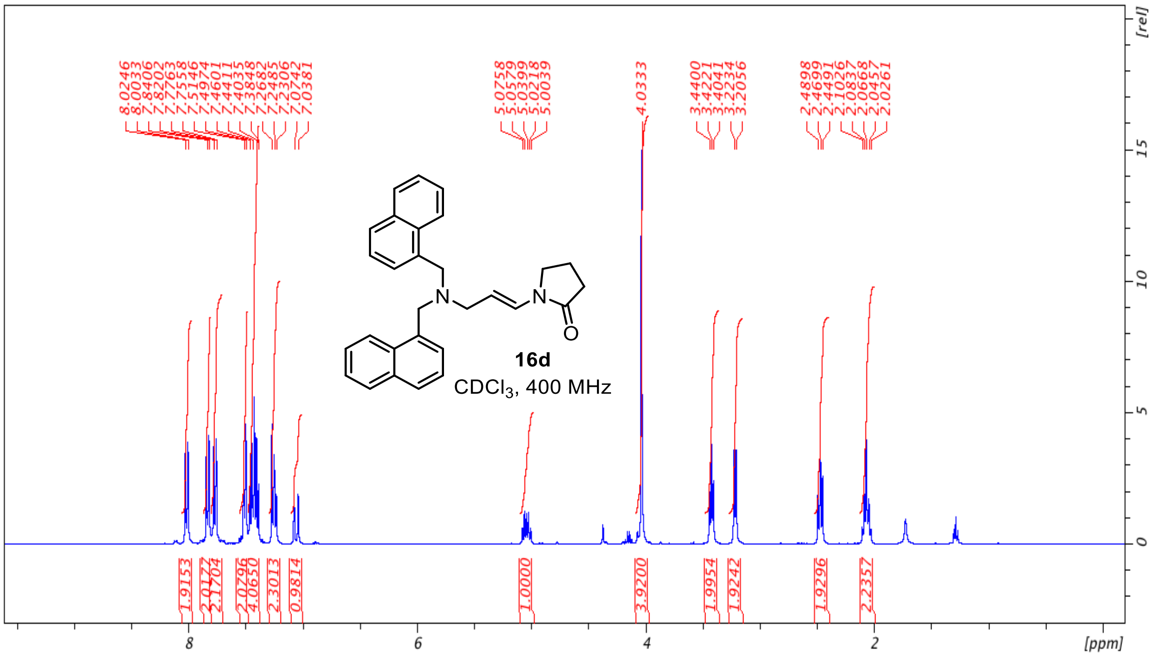

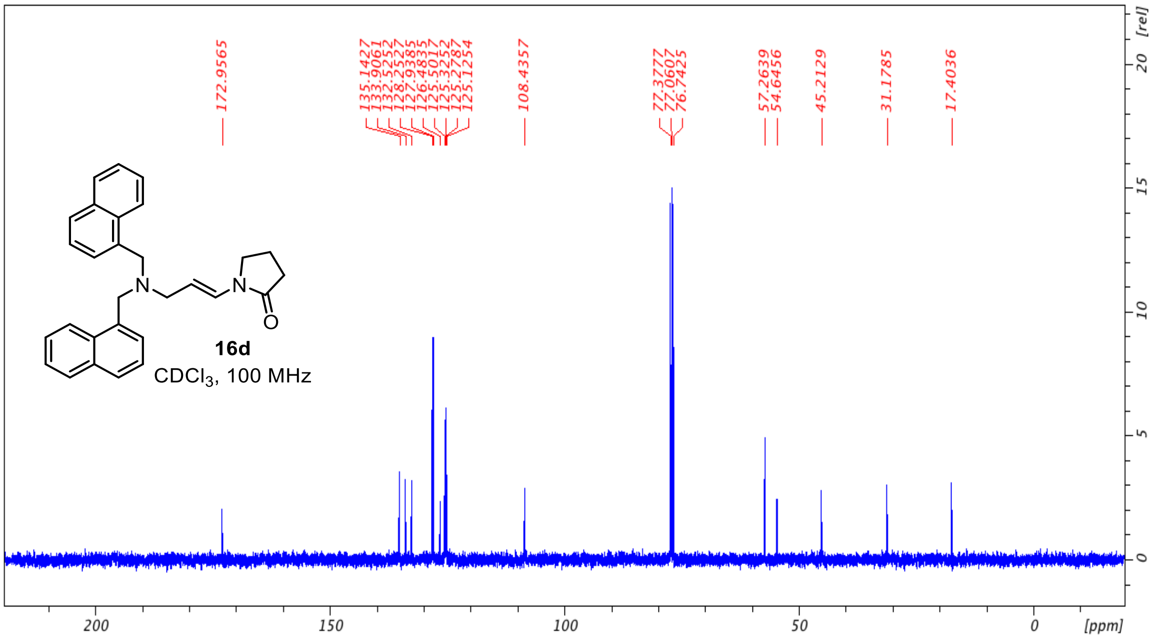

Compound **16e**

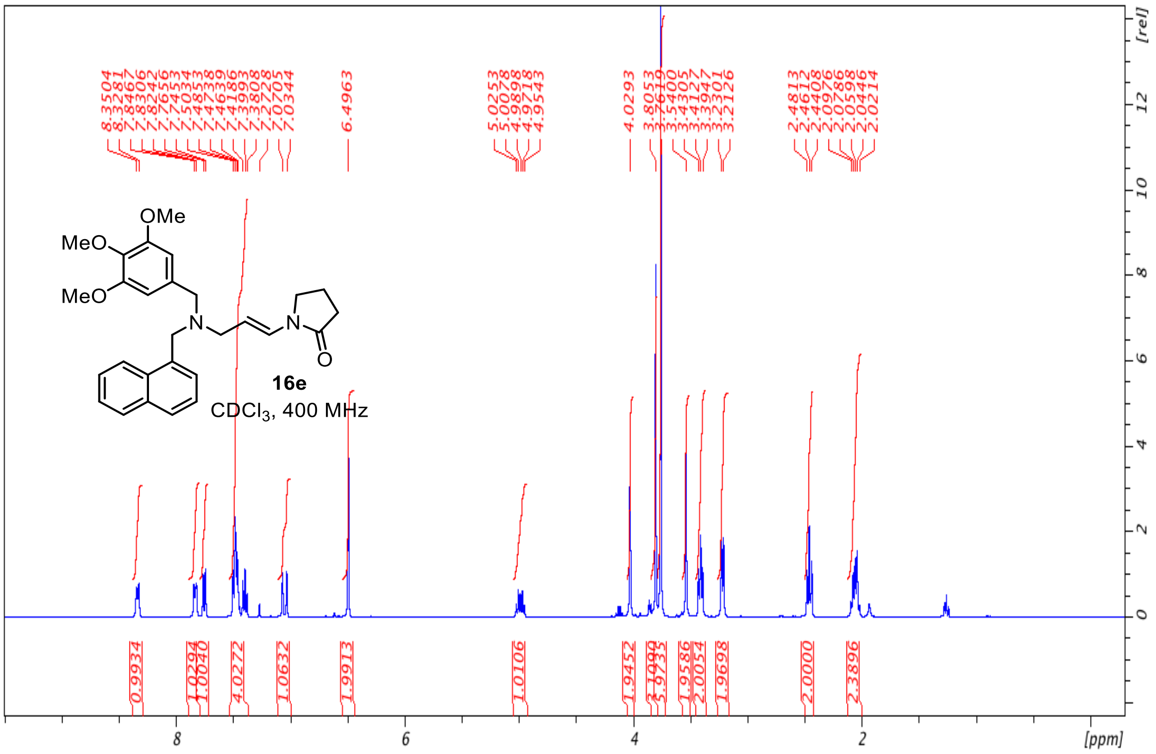

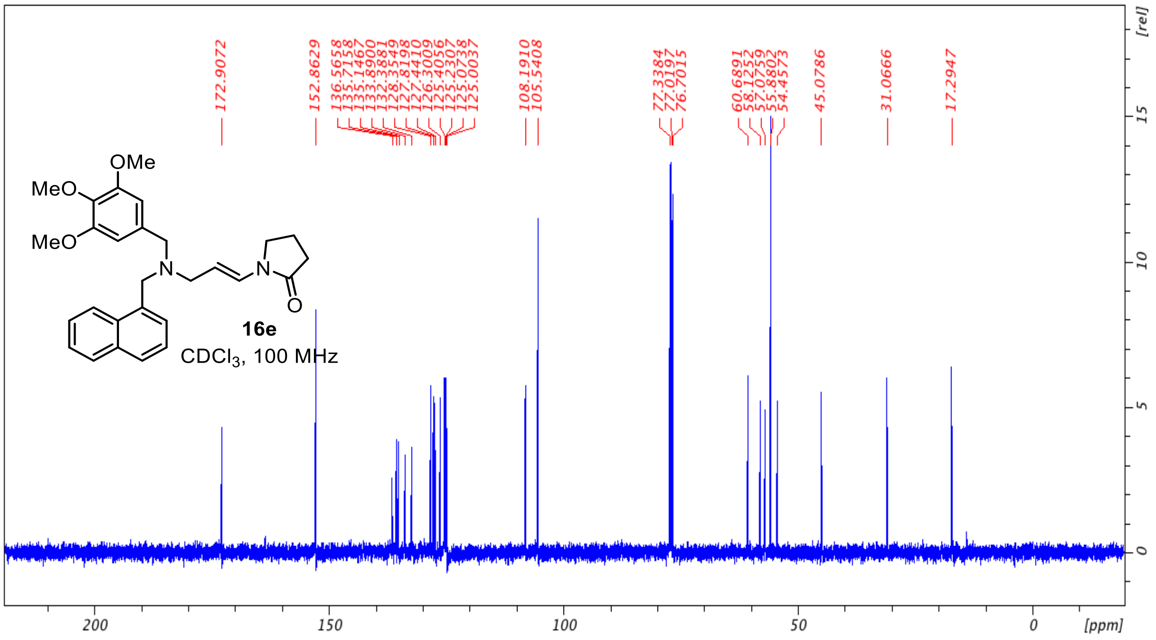

Compound **16f**

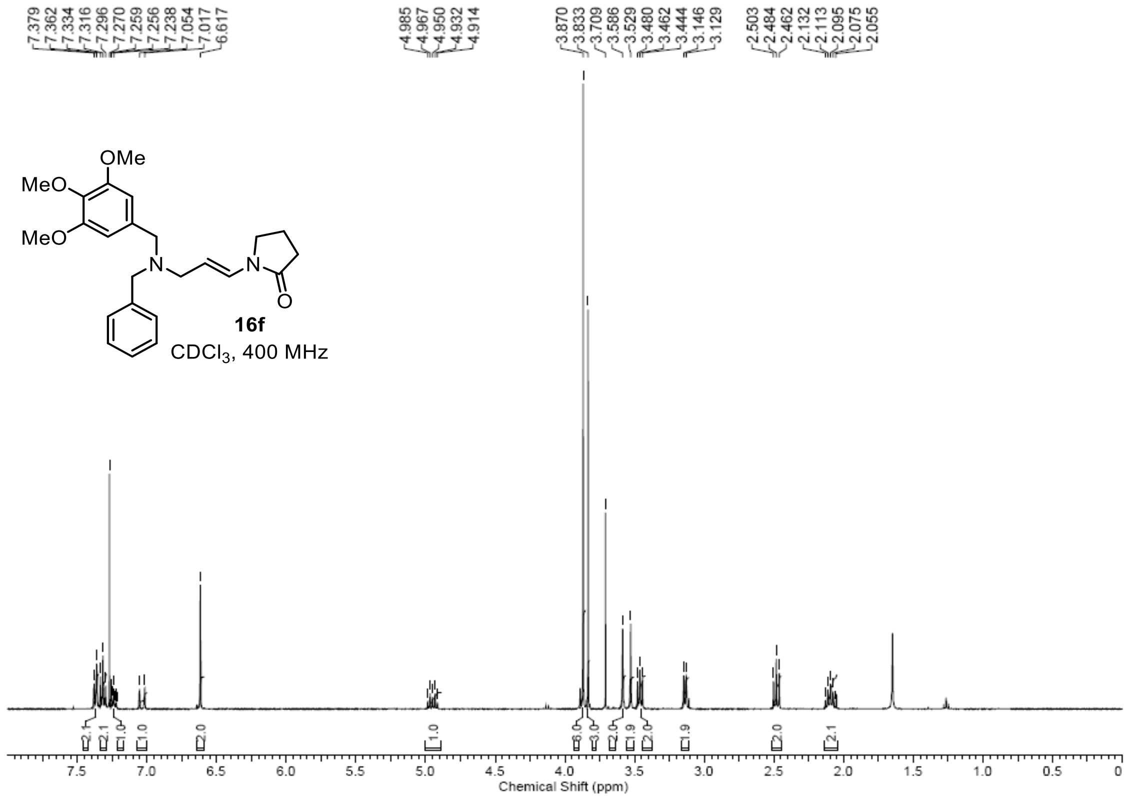

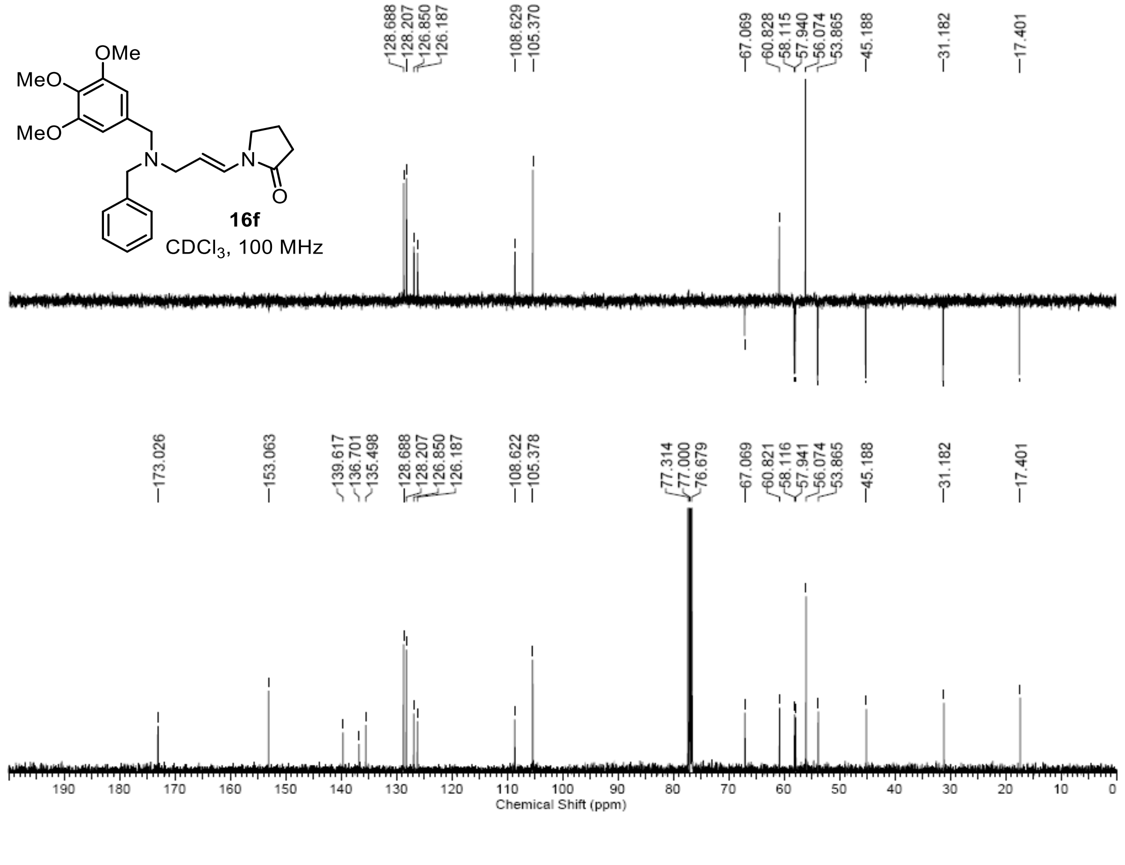

Compound **16g**

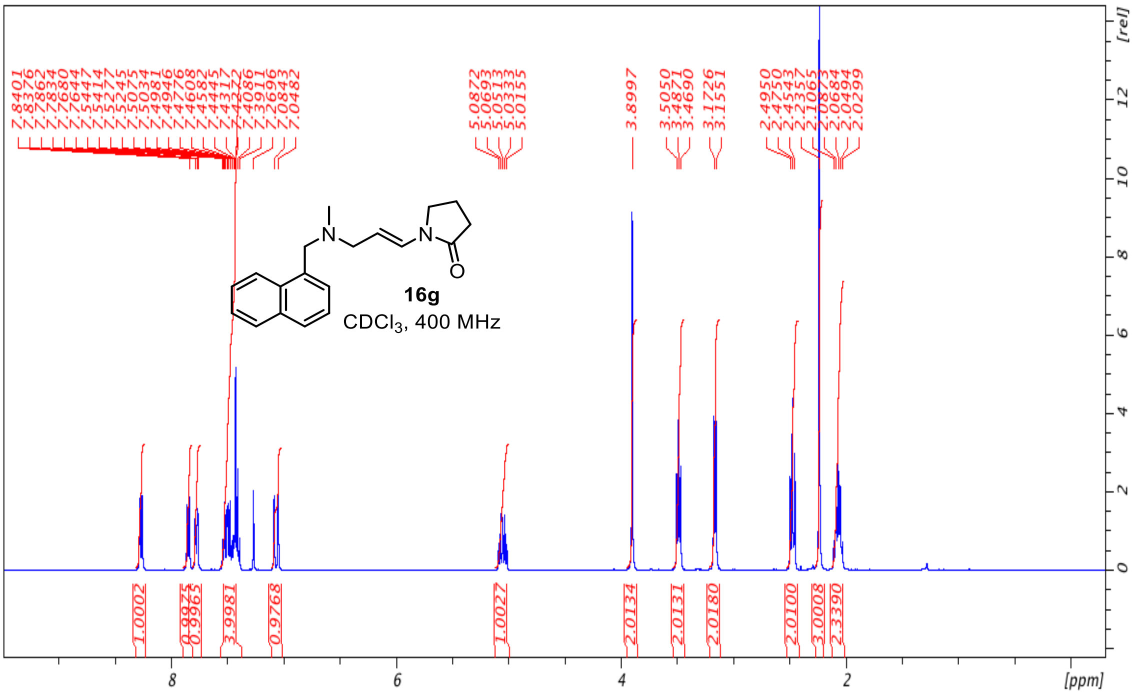

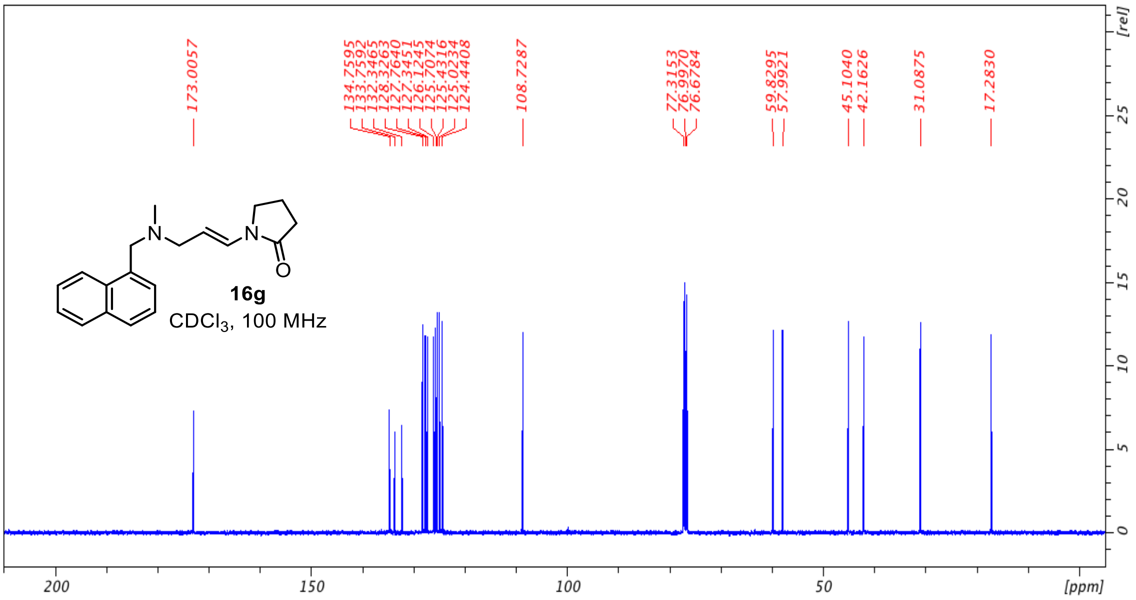

Compound **16h**

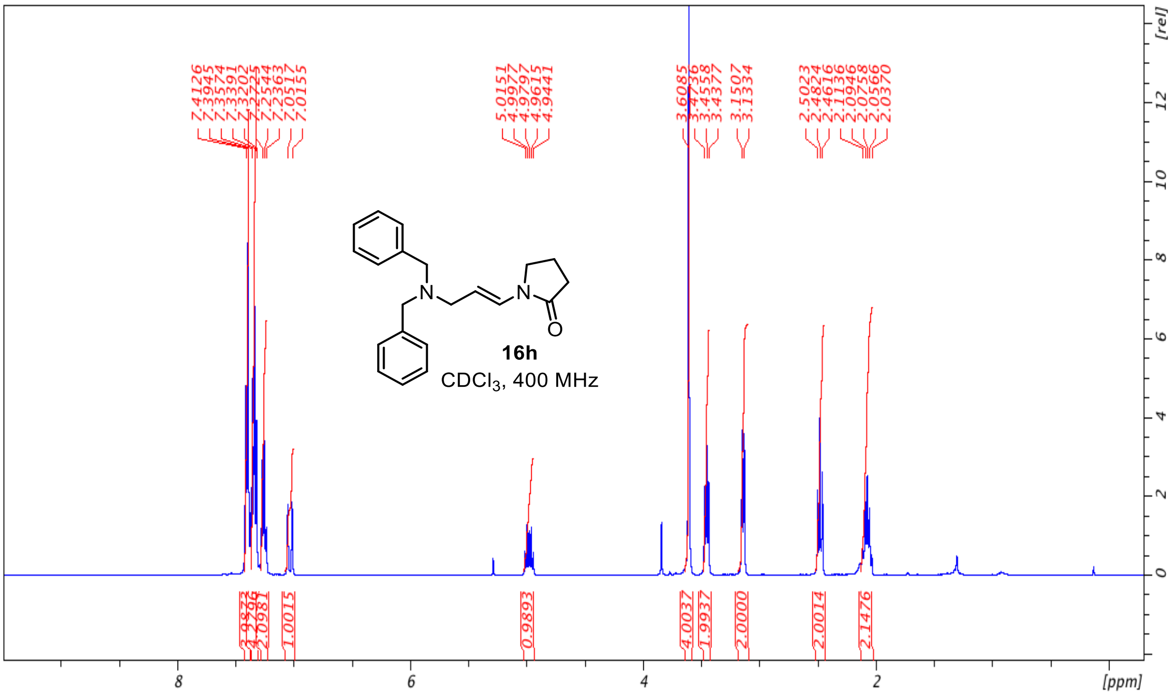

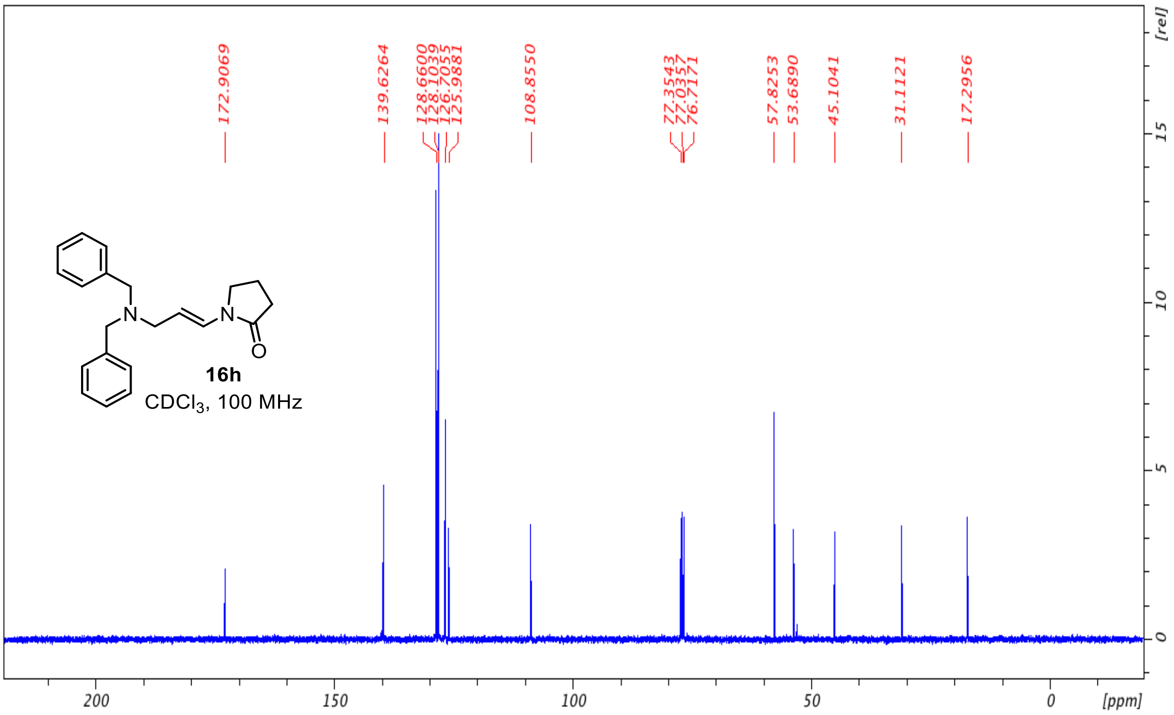

Compound **16i**

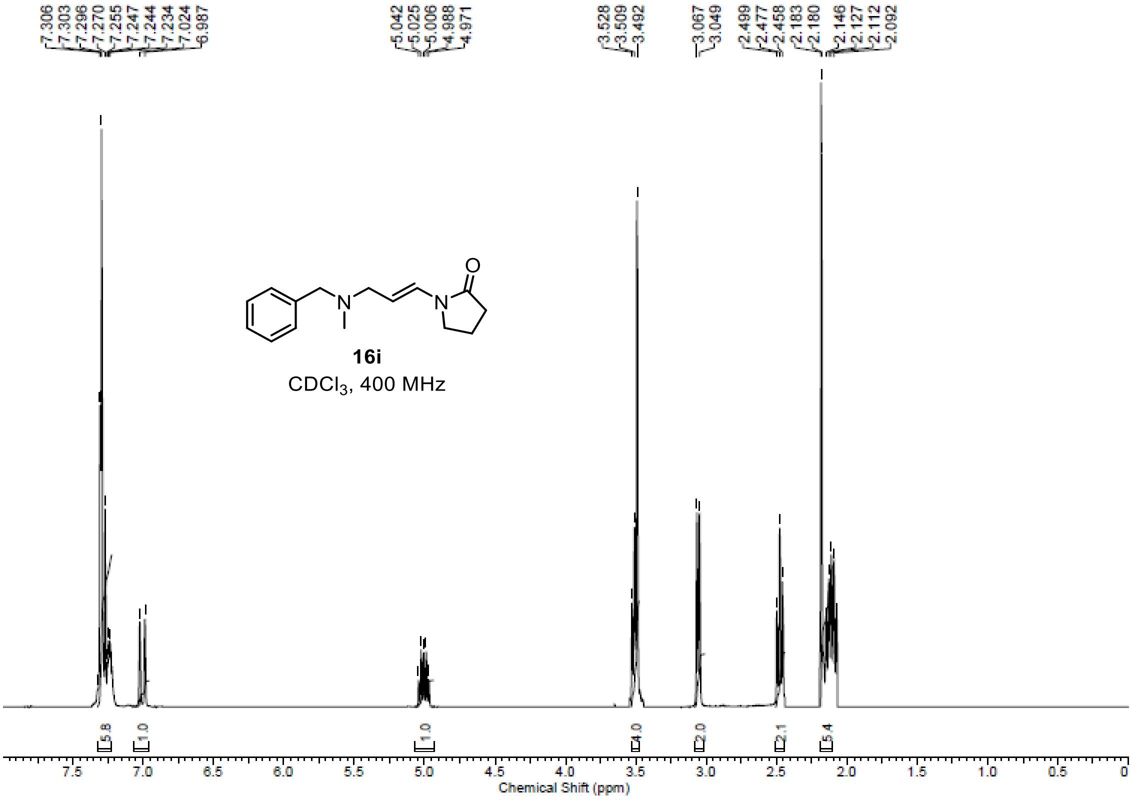

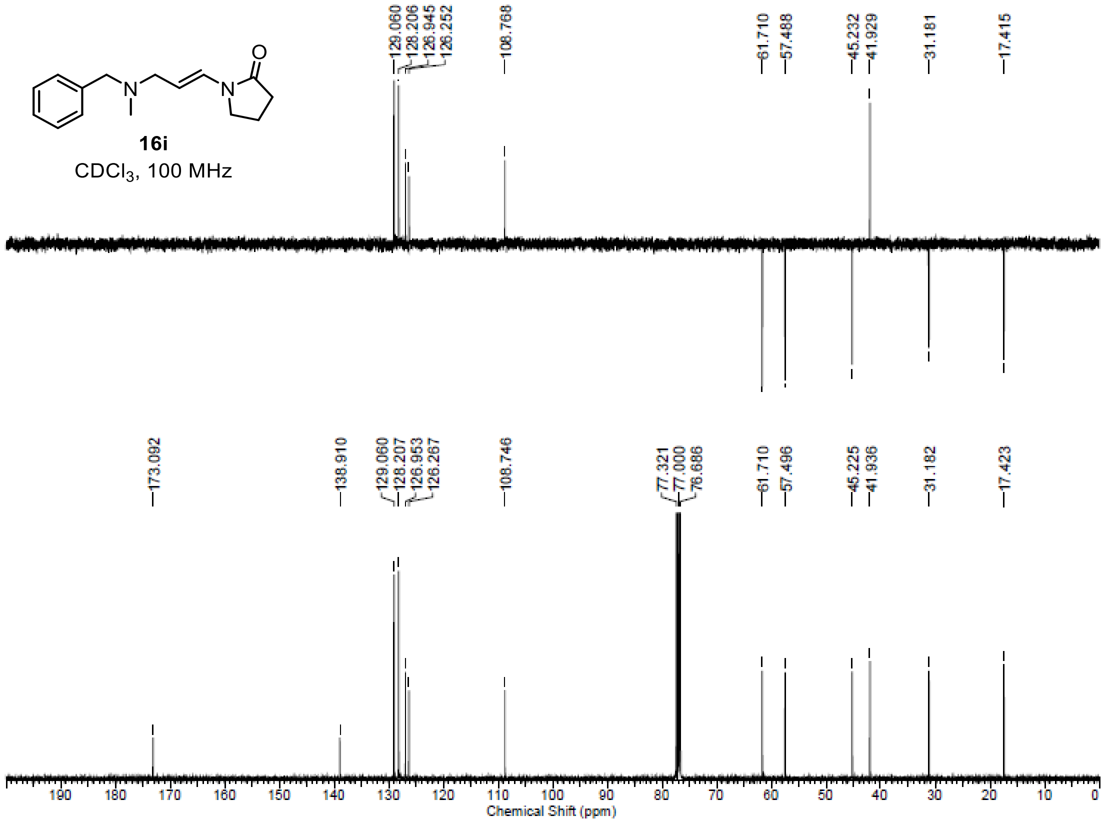

Compound **16k**

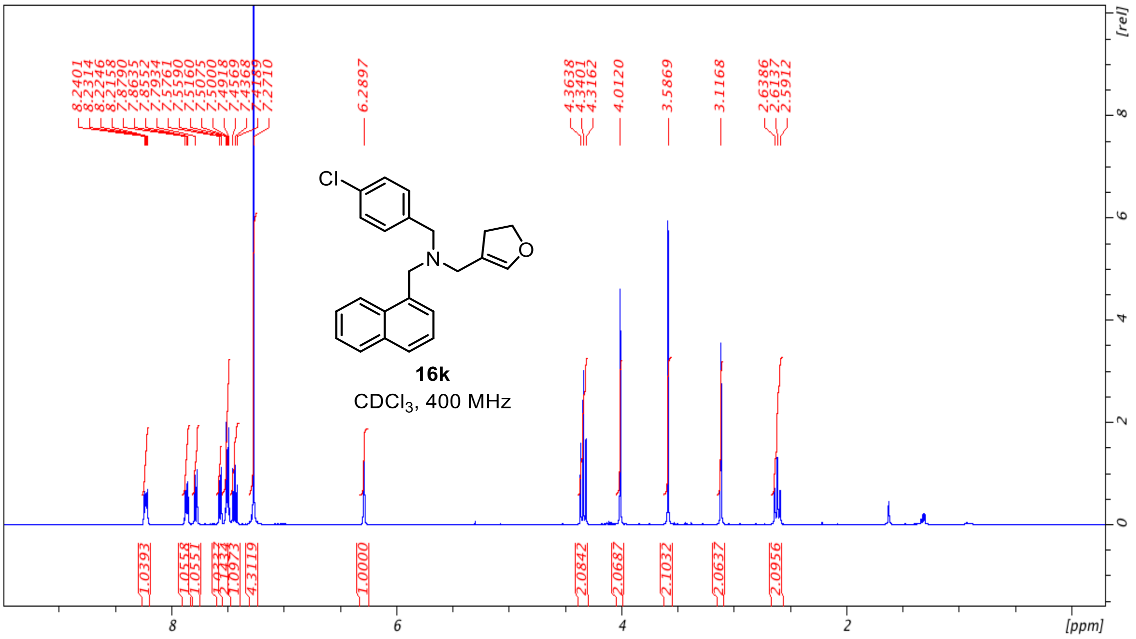

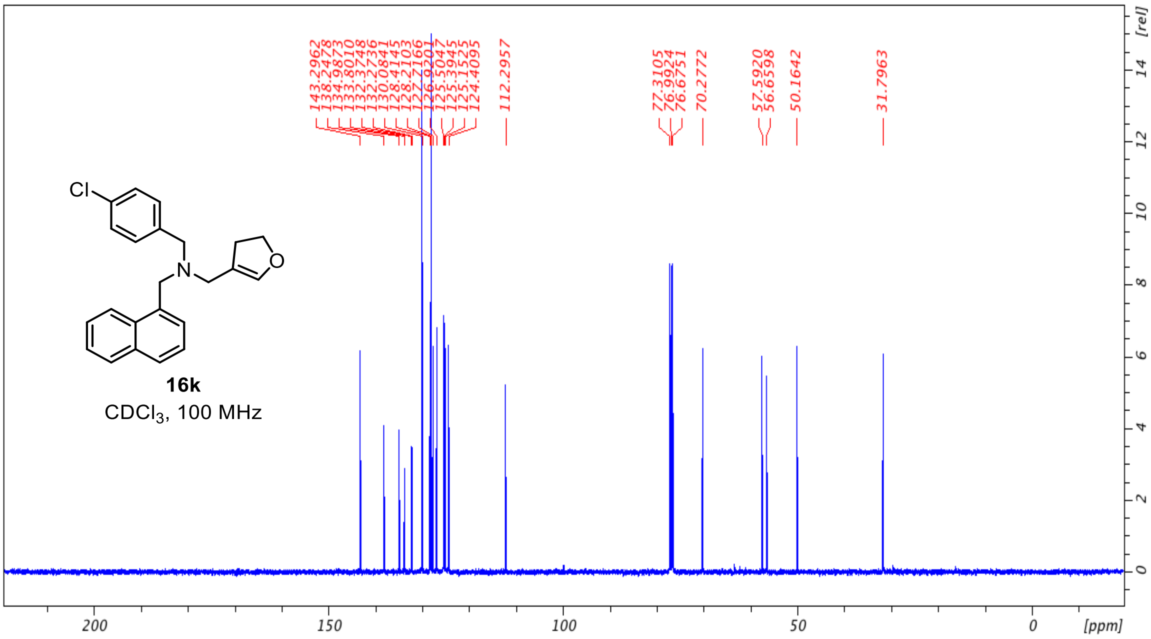

Naftifine

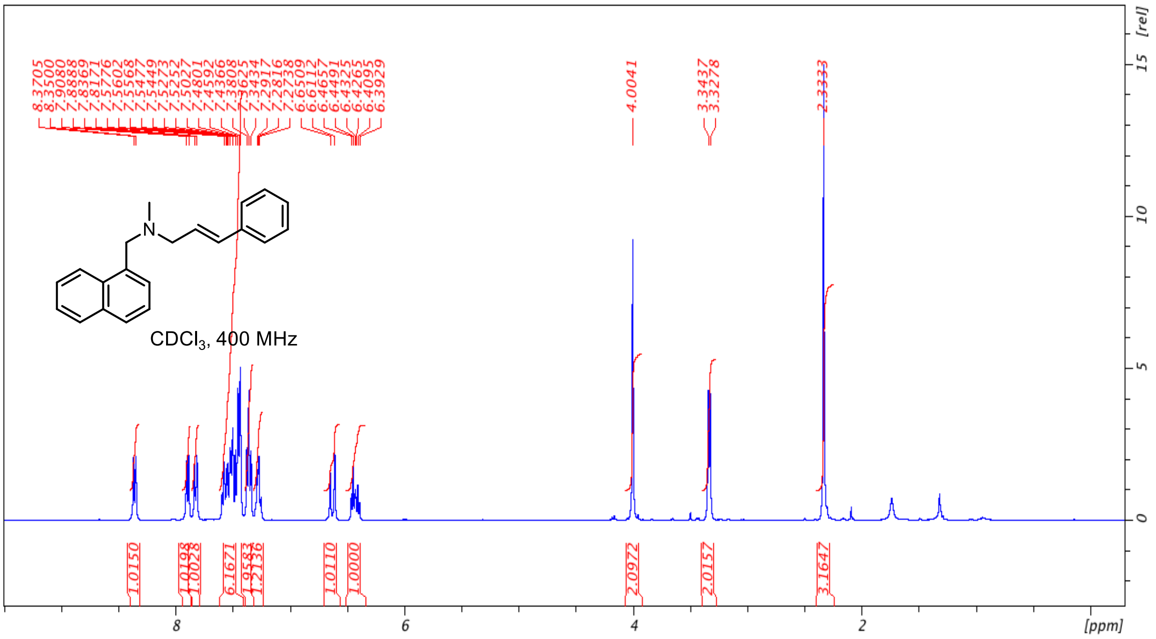

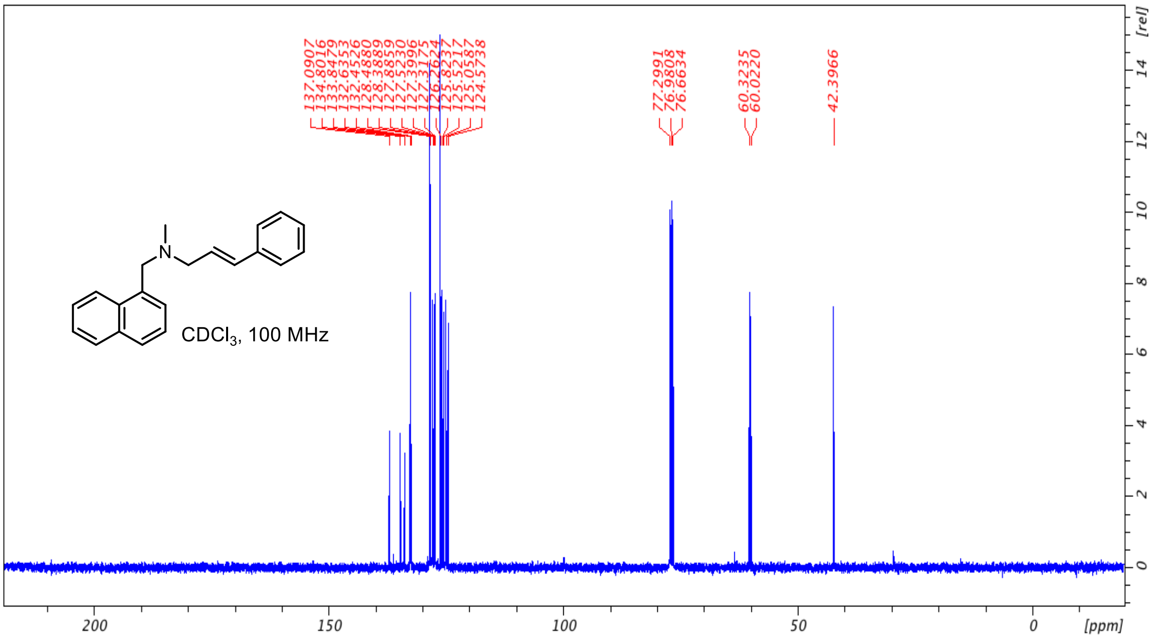

Compound **21b**

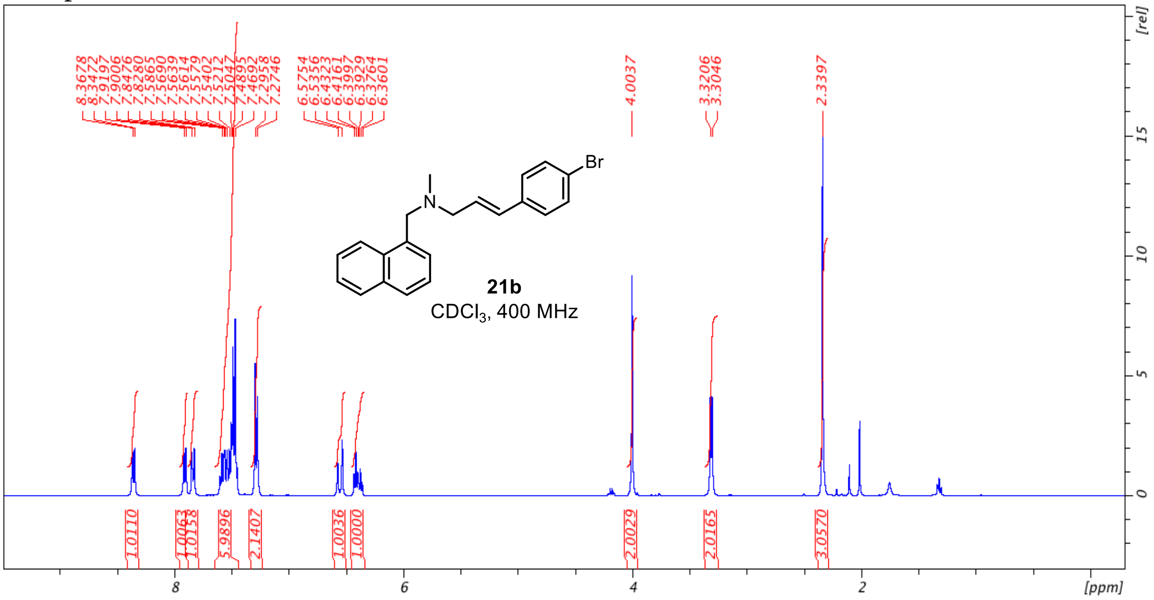

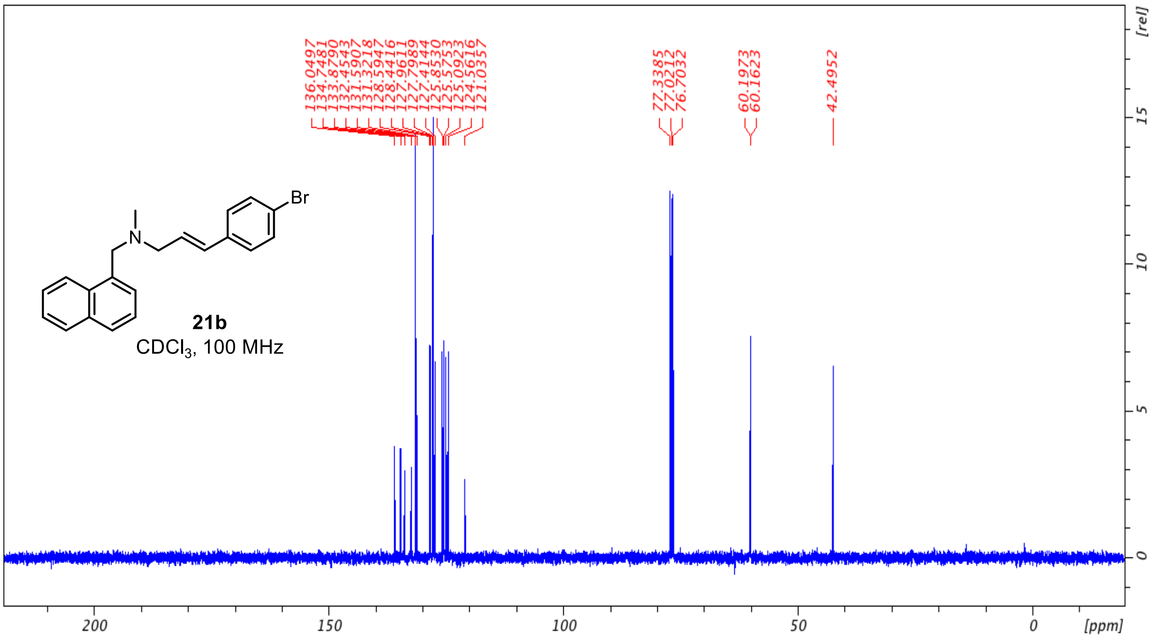

Compound **21c**

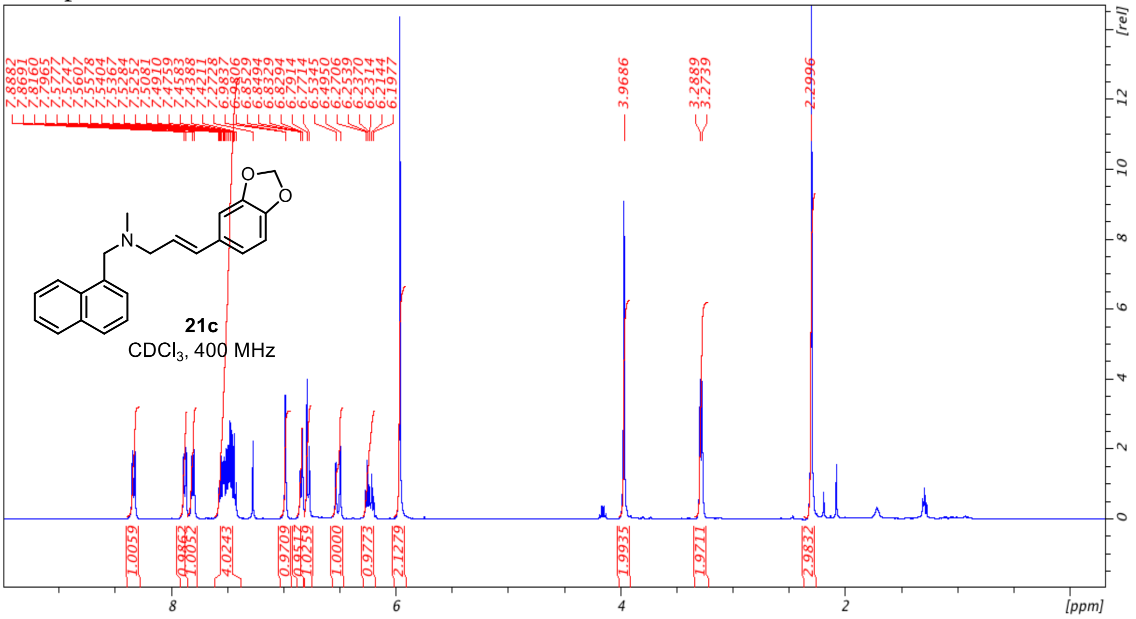

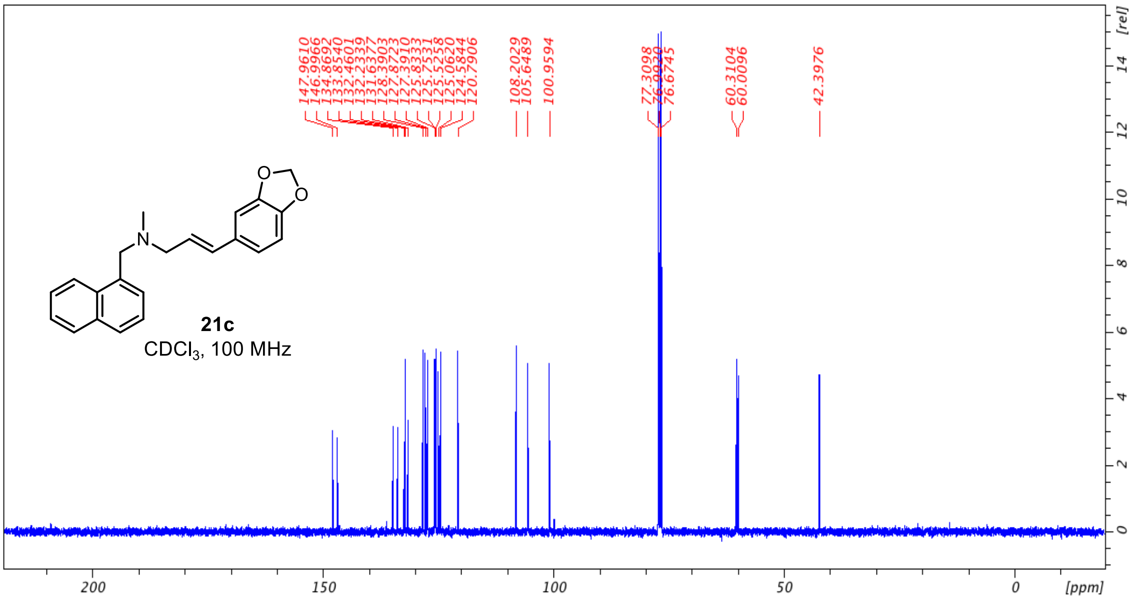

Compound **21d**

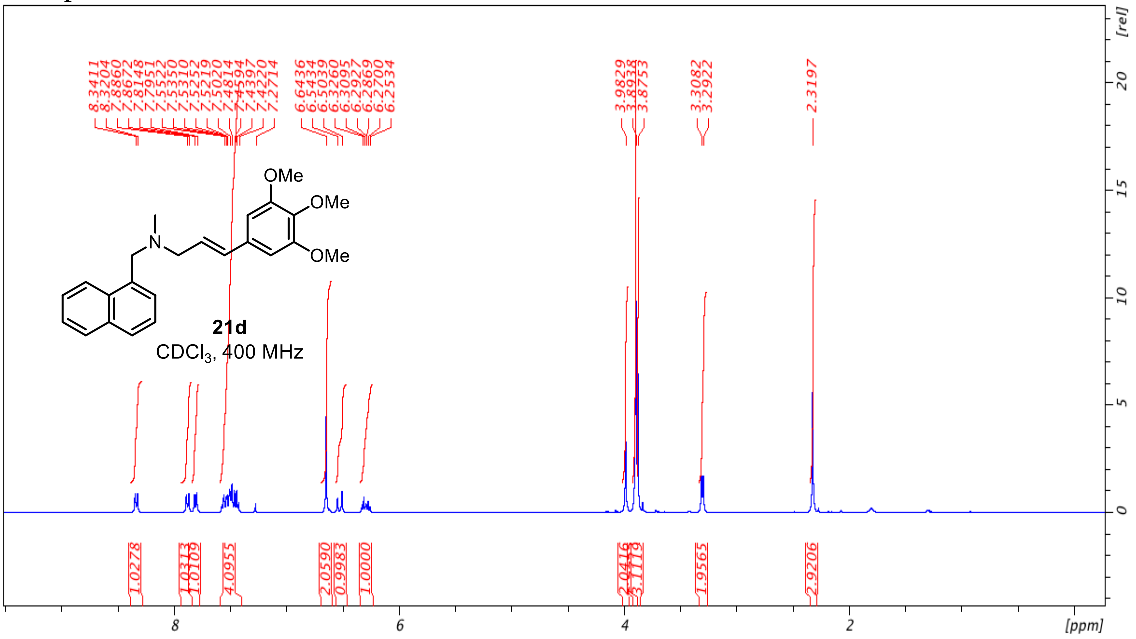

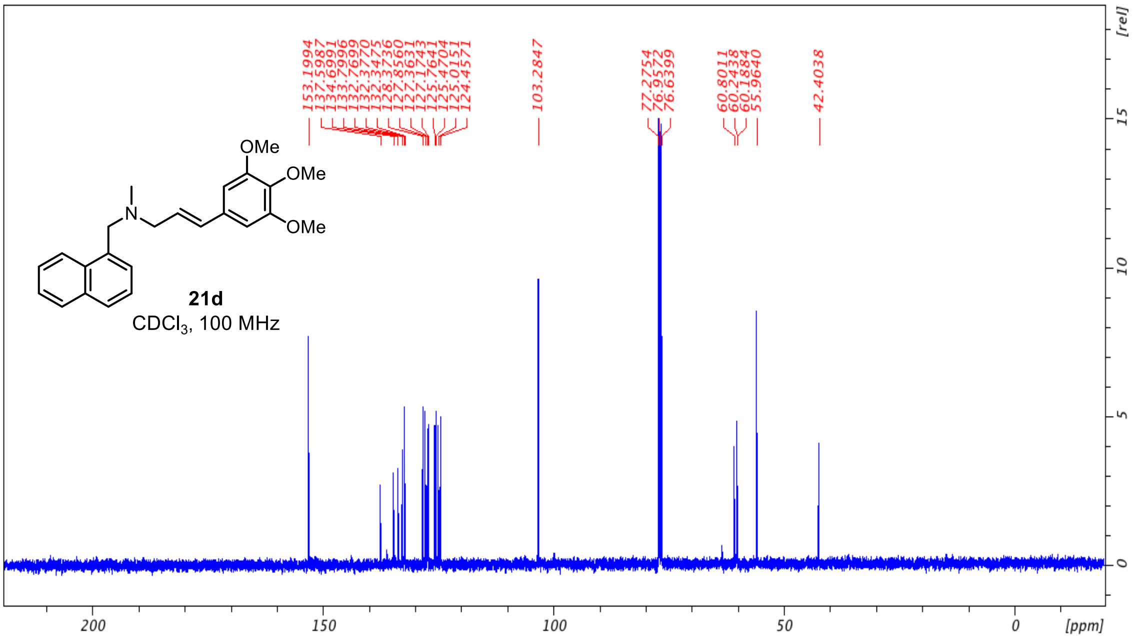

Compound **21e**

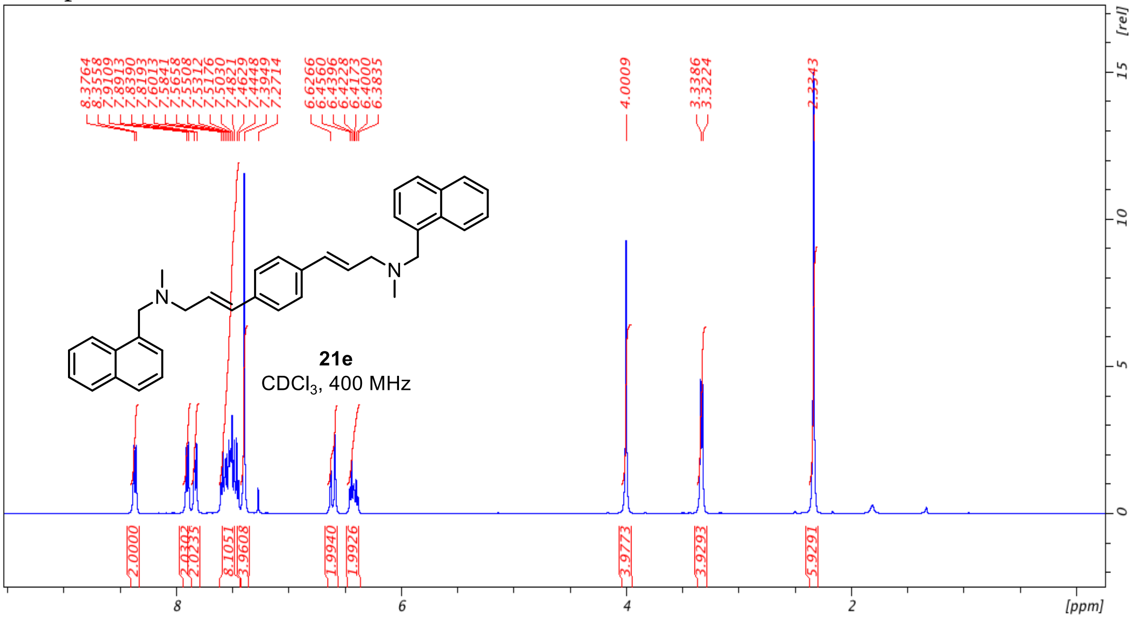

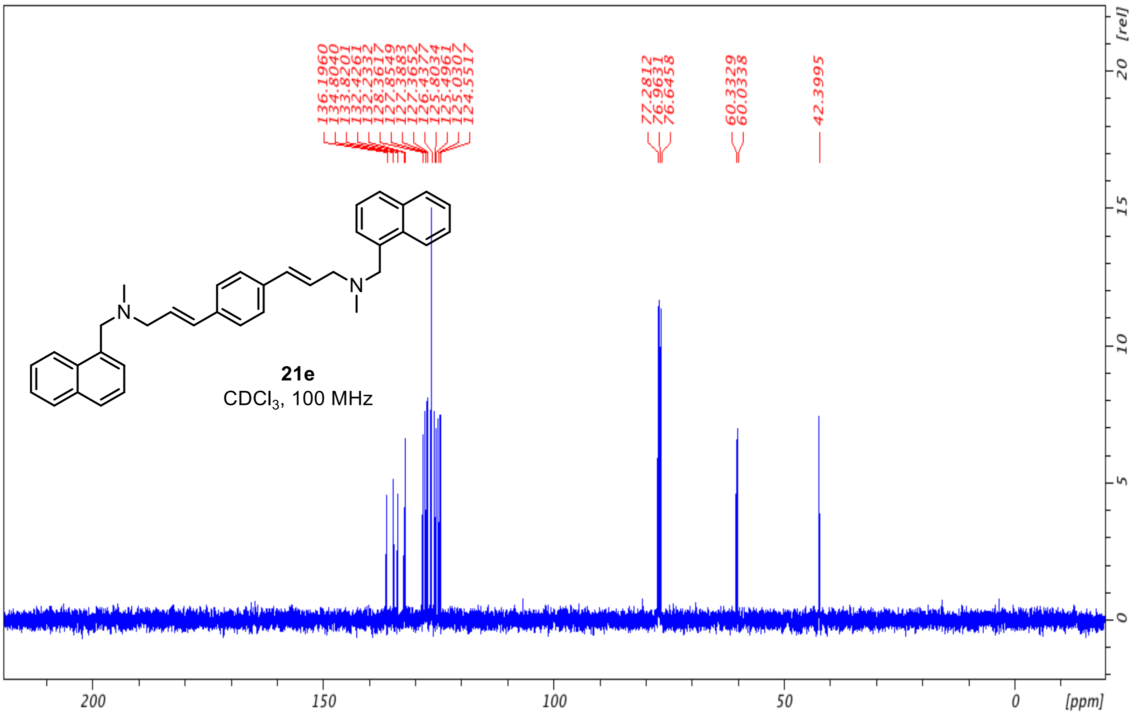

Compound **22a**

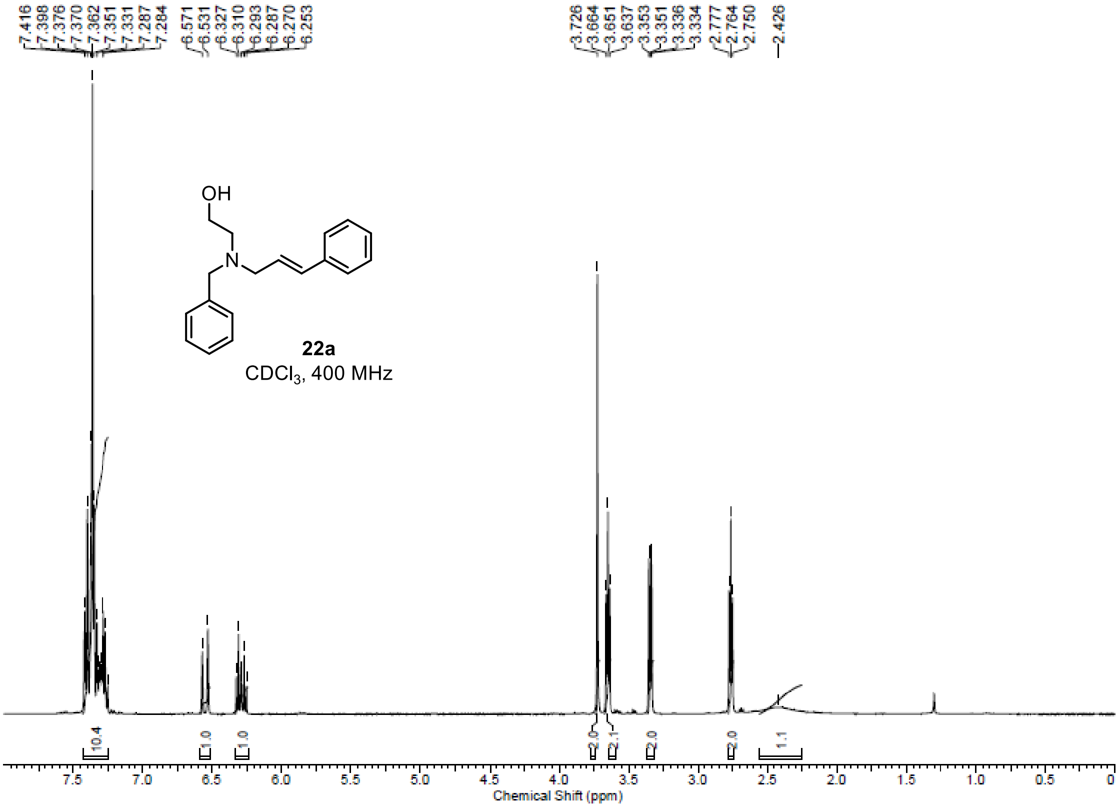

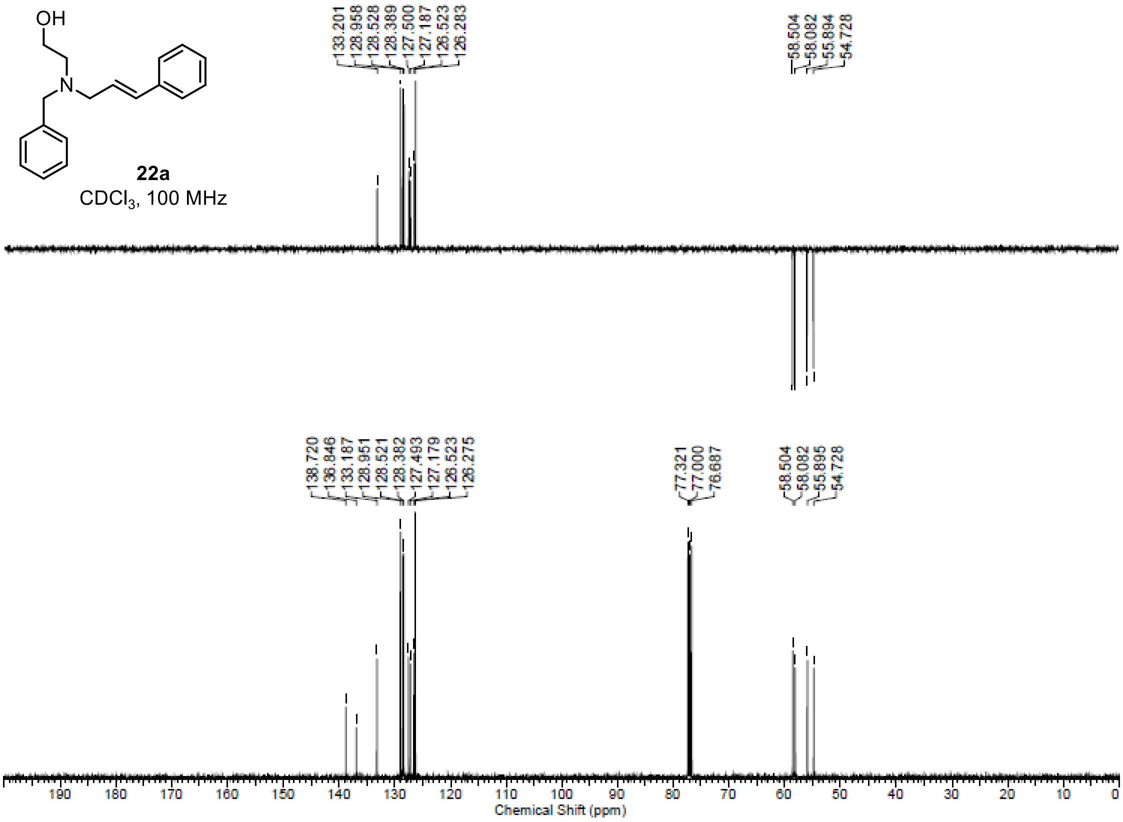

Compound **22f**

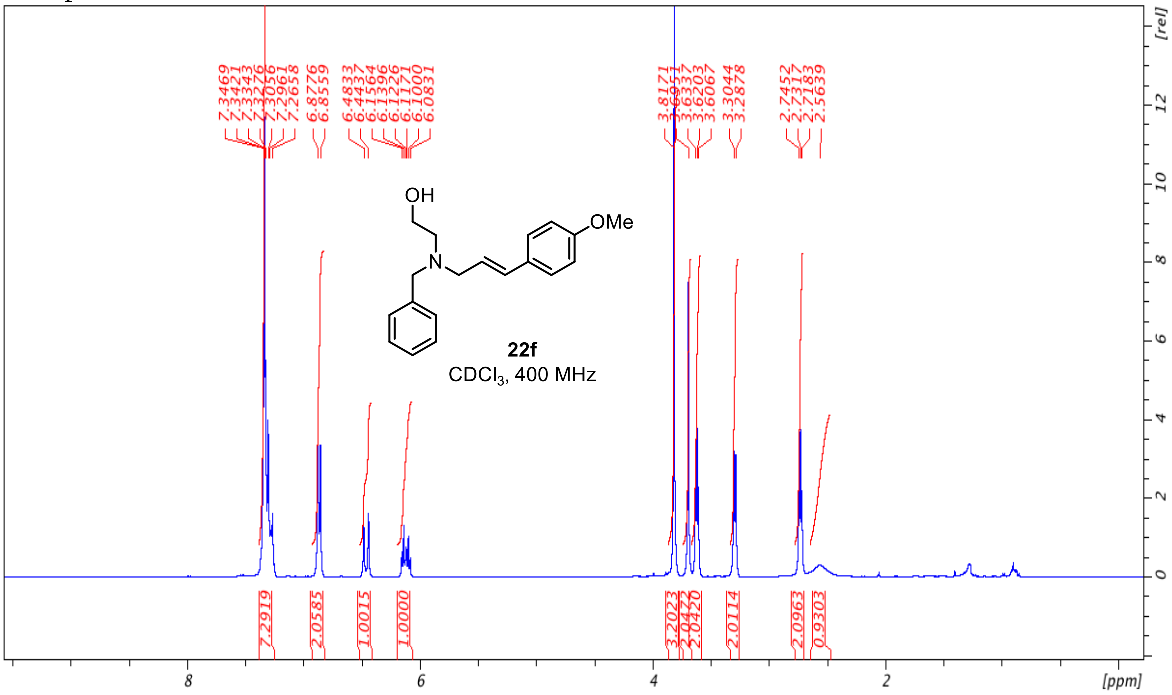

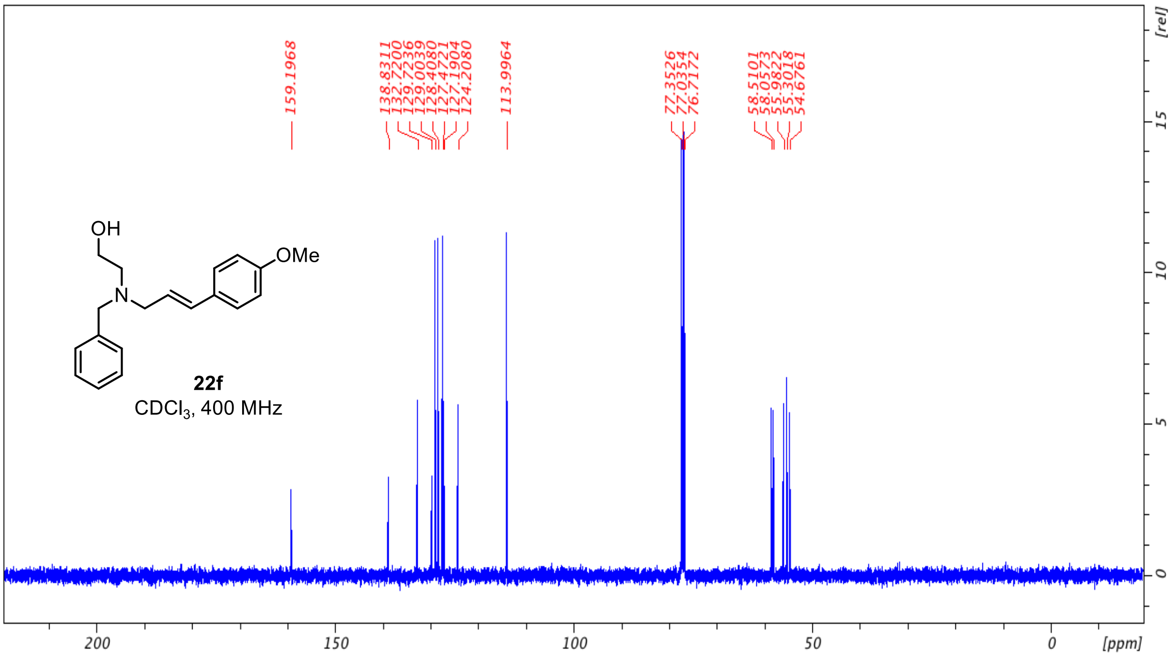

Supplement: Supplementary file 1 [file molecules-23-00520-s001.pdf]
